# Flavylium-Based Hypoxia-Responsive Probe for Cancer Cell Imaging

**DOI:** 10.3390/molecules26164938

**Published:** 2021-08-15

**Authors:** Thitima Pewklang, Sirawit Wet-osot, Sirilak Wangngae, Utumporn Ngivprom, Kantapat Chansaenpak, Chuthamat Duangkamol, Rung-Yi Lai, Parinya Noisa, Mongkol Sukwattanasinitt, Anyanee Kamkaew

**Affiliations:** 1 School of Chemistry, Institute of Science, Suranaree University of Technology, Nakhon Ratchasima 30000, Thailand; thitima.27.1996@gmail.com (T.P.); sirawitwetosot@gmail.com (S.W.-o.); swsirilak00@gmail.com (S.W.); utumporn_n@kkumail.com (U.N.); chuthamat_duangkamol@hotmail.com (C.D.); 2 National Nanotechnology Center, National Science and Technology Development Agency, Thailand Science Park, Pathum Thani 12120, Thailand; kantapat.cha@nanotec.or.th; 3 Laboratory of Cell-Based Assays and Innovations, Institute of Agricultural Technology, School of Biotechnology, Suranaree University of Technology, Nakhon Ratchasima 30000, Thailand; p.noisa@sut.ac.th; 4 Thailand Nanotec-CU Center of Excellence on Food and Agriculture, Department of Chemistry, Faculty of Science, Chulalongkorn University, Bangkok 10330, Thailand; mongkol.s@chula.ac.th

**Keywords:** flavylium, azo dye, hypoxia detection, turn-on fluorescent sensor, activity-based sensing

## Abstract

A hypoxia-responsive probe based on a flavylium dye containing an azo group (**AZO-Flav**) was synthesized to detect hypoxic conditions via a reductase-catalyzed reaction in cancer cells. In in vitro enzymatic investigation, the azo group of **AZO-Flav** was reduced by a reductase in the presence of reduced nicotinamide adenine dinucleotide phosphate (NADPH) followed by fragmentation to generate a fluorescent molecule, **Flav-NH_2_**. The response of **AZO-Flav** to the reductase was as fast as 2 min with a limit of detection (LOD) of 0.4 μM. Moreover, **AZO-Flav** displayed high enzyme specificity even in the presence of high concentrations of biological interferences, such as reducing agents and biothiols. Therefore, **AZO-Flav** was tested to detect hypoxic and normoxic environments in cancer cells (HepG2). Compared to the normal condition, the fluorescence intensity in hypoxic conditions increased about 10-fold after 15 min. Prolonged incubation showed a 26-fold higher fluorescent intensity after 60 min. In addition, the fluorescence signal under hypoxia can be suppressed by an electron transport process inhibitor, diphenyliodonium chloride (DPIC), suggesting that reductases take part in the azo group reduction of **AZO-Flav** in a hypoxic environment. Therefore, this probe showed great potential application toward in vivo hypoxia detection.

## 1. Introduction

Solid tumor growth is restricted by vascularization, which requires oxygen and nutrient supply. It has been reported that the median oxygen concentration is around 4% in some solid tumors and can be decreased to as low as 0% in a certain area [1,2]. Such low oxygen conditions in tumors are known as hypoxia, which is primarily due to variations in microcirculation and temporary disturbance in oxygen perfusion [3]. Tumor hypoxia usually occurs at a distance of 100–200 μm from blood vessels and seems to be strongly associated with tumor propagation, malignant progression and resistance to chemo- and radiotherapy [4,5]. Hypoxia could regulate the expression of several genes by the stabilization of hypoxia-inducible factor 1α (HIF-1α), leading to various biological phenomena [6]. Thus, the detection of hypoxia is an important approach to investigate its biological effects.

In the past decade, several activity-based fluorescent probes for hypoxia sensing have been developed and tested in living cells [7,8,9,10]. Various functional groups, such as aromatic nitro, azo, and quinone groups, were reported as hypoxia-sensitive moieties; their signaling mechanisms rely on a photoinduced electron transfer (PeT) [11]. However, most of these probes are photo-unstable, have low selectivity, and are susceptible to the pH or polarity of the media [1,12,13]. Hence, a better chemical- and photo-stable probe with high selectivity and sensitivity is still required to be developed for hypoxia detection.

Compared to the normal environment, many endogenous cytochrome P450 enzymes are highly expressed in hypoxic locations [12]. Therefore, many prodrugs were designed to be activated by oxidation and catalyzed by cytochrome P450 enzymes [13,14,15,16]. Moreover, due to the activation of cytochrome P450 enzymes requiring reductases to transfer electrons to reduce their iron centers, cytochrome P450 reductases are also more present in cancer cells than normal cells [17]. Hence, cytochrome P450 reductases are alternative targets in cancer research [18,19]. Since cytochrome P450 reductases catalyze electron transfer to activate cytochrome P450 enzymes, few functional groups susceptible to reduction have been applied in probe design [20,21]. Notably, the azo aromatic compounds were reported to be good substrates for cytochrome P450 reductases and some azo-containing fluorescent probes displayed effective results in hypoxia detection [7,22,23,24].

**Scheme 1:**
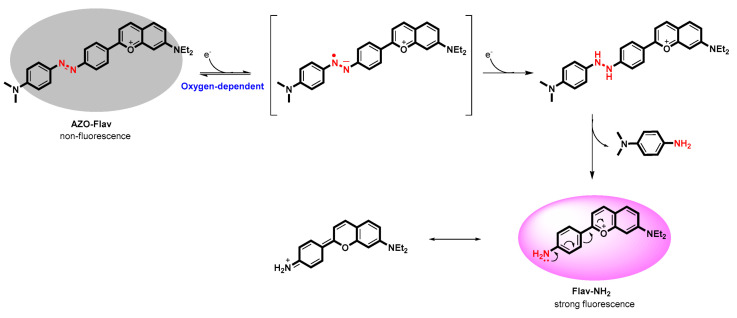
Proposed activation mechanism of **AZO-Flav** reduced by a reductase under hypoxia conditions in this work.

In this study, we designed an azo-flavylium probe to detect cancer cells, because flavylium structures could be derivatized to possess interesting photophysical properties [25,26]. For example, a flavylium structure was modified to contain a nitroaromatic ring for the detection of nitroreductase activity in living cells by observing its ratiometric fluorescence changes [27]. A similar strategy was applied on the flavylium design to probe hydrogen polysulfide (H_2_S*_n_*), which reduced the nitroaromatic group of the probe to the corresponding amino group showing 87-fold fluorescence enhancement [28]. However, there has been no attempt to incorporate an azo moiety in a flavylium dye for controllable fluorescent off/on switching for hypoxia detection. Therefore, we designed and synthesized a flavylium dye containing an azo group (**AZO-Flav**) as a fluorescent turn-on probe for hypoxia response in cancer cells. The characterization of **AZO-Flav** showed negligible fluorescence due to the azo entity, a photoisomerizable quenching unit. However, after the reductase-catalyzed reaction the fluorescence was distinctively enhanced. This is because the azo group was reduced followed by the elimination of 4-dimethylaminoaniline to generate the fluorescent molecule, **Flav-NH_2_** (Scheme 1). Furthermore, the probe displayed favorable photophysical properties, excellent stability, and high selectivity toward hypoxia detection. Lastly, **AZO-Flav** was applied to detect cancer cells (HepG2) in hypoxic conditions compared with normoxic conditions.

## 2. Results and Discussion

### 2.1. Probe Synthesis and Characterization

To develop an activity-based fluorescent probe for hypoxia detection, the key is to incorporate a specific reactive unit. In our design, we integrated an azo group into the flavylium skeleton as a reactive unit for reductase-catalyzed reduction. Furthermore, the incorporation of an azo group was aimed to block the dye’s fluorescence. In general, the spectroscopic properties of azobenzene have been reported to be a non-fluorogenic compound due to ultrafast isomerization of the azo bond (-N=N-) after photoexcitation [29,30]. Therefore, we hypothesized that after cleavage of the azo unit, the fluorescence of the flavylium dye would be restored.

**Scheme 2:**
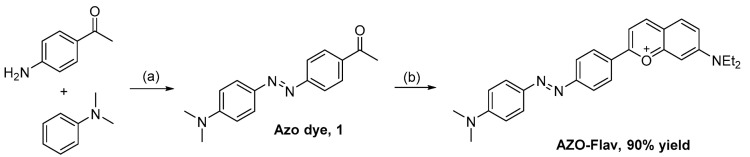
Synthetic scheme for **AZO-Flav**; (a) 3M HCl, NaNO_2_, AcOH, 0 °C, 2 h. (b) 4-(diethylamino)salicylaldehyde, H_2_SO_4_, 90 °C, 2 h.

A novel azo-flavylium dye (**AZO**-**Flav**) was synthesized according to Scheme 2. First, the azo dye **1** was obtained through the diazotization reaction of 4-aminoacetophenone and *N,N*-dimethylaniline. Next, the condensation between the azo dye **1** and 4-(diethylamino)salicylaldehyde under acid conditions generated the corresponding product, **AZO**-**Flav**, in a yield of 90%. In addition, the proposed product, **Flav-NH_2_**, in Scheme 1 was synthesized according to the literature [31]. The detailed synthesis and characterization of **AZO-flav** and **Flav-NH_2_** are presented in the Supporting Information.

### 2.2. Photophysical Properties of Probe **AZO-Flav** and Fluorophore **Flav-NH_2_**

**Figure 1:**
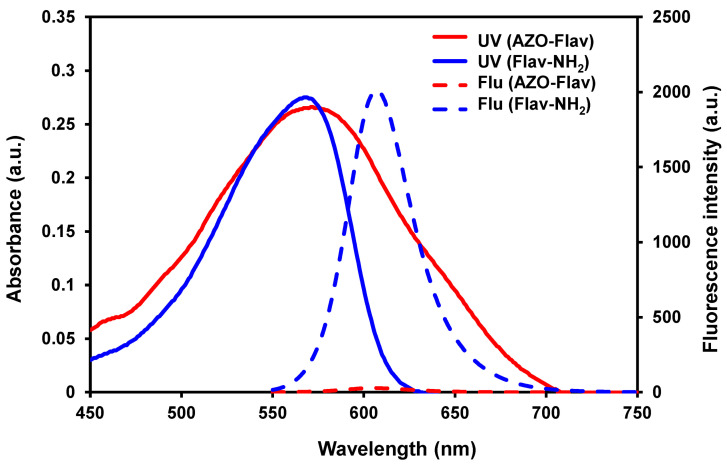
UV-vis-NIR absorption and fluorescence spectra of **AZO-Flav** and **Flav-NH_2_** (10 μM) in 100 mM phosphate buffer (pH 7.4) excited at 540 nm.

The photophysical properties of the azo probe, **AZO-Flav**, and the flavylium fluorophore, **Flav-NH_2_**, were investigated to confirm the alteration of the fluorescence process after the fluorophore incorporated with the azo group. The UV-Vis-NIR absorption and fluorescent emission spectra of **AZO-Flav** and **Flav-NH_2_** (10 μM) in 100 mM phosphate buffer (pH 7.4) are shown in Figure 1. **AZO-Flav** displays a broader absorption peaking around 570 nm while **Flav-NH_2_** exhibits narrower absorption band peaking around 560 nm. In their emission profiles, negligible fluorescence was observed from **AZO-Flav** due to the depletion of absorbed energy by isomerization of the azo bond in which a similar phenomenon occurred in the reported azo-containing dyes [11]. On the other hand, the flavylium fluorophore (**Flav-NH_2_**) displays strong fluorescence peaking at 607 nm, which is also concentration dependent (Figure S1). These emission profiles showed the great difference of emission intensities between **AZO-Flav** and **Flav-NH_2_**. Therefore, **Flav-NH_2_** generated from **AZO-Flav** reduction (Scheme 1) could be an excellent turn-on indicator for hypoxia detection.

### 2.3. Fluorescence Stability towards pH Changes

**Figure 2:**
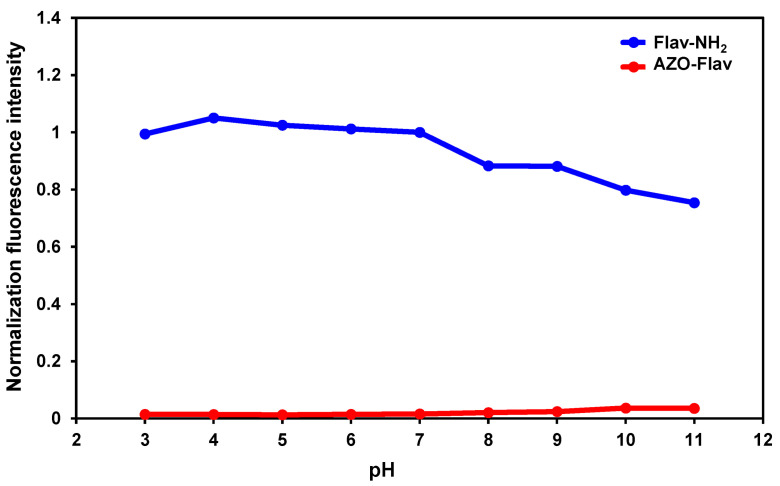
Fluorescent response of **AZO-Flav** and **Flav-NH_2_** (10 μM) at different pH values (λ_ex_ = 540 nm and λ_em_ = 607 nm).

To ensure our probe can effectively detect the oxygen deficiency area in a tumor, the pH sensitivities of **AZO-Flav** and **Flav-NH_2_** were tested and monitored by fluorescence spectroscopy. The results showed that the fluorescence signals of the reduction product (**Flav-NH_2_**) were quite stable in the acidic to neutral pH range (pH 3–7), whereas the emission intensity dropped about 10–20% in the basic solution (pH 8–11). On the other hand, **AZO-Flav** still showed low fluorescence signals in pH ranging from 3 to 11 (Figure 2 and Figure S2), implying no cleavage of the azo bond by altering the pH. These results suggested that the probe **AZO-Flav** could be stable in hypoxic zones, which are usually acidic [32]. Moreover, the product (**Flav-NH_2_**) from the reduction reaction could still maintain its full fluorescent intensity in acidic condition.

### 2.4. In Vitro Reduction of **AZO-Flav** by E. coli Flavodoxin Reductase (EcFldR)

**Figure 3:**
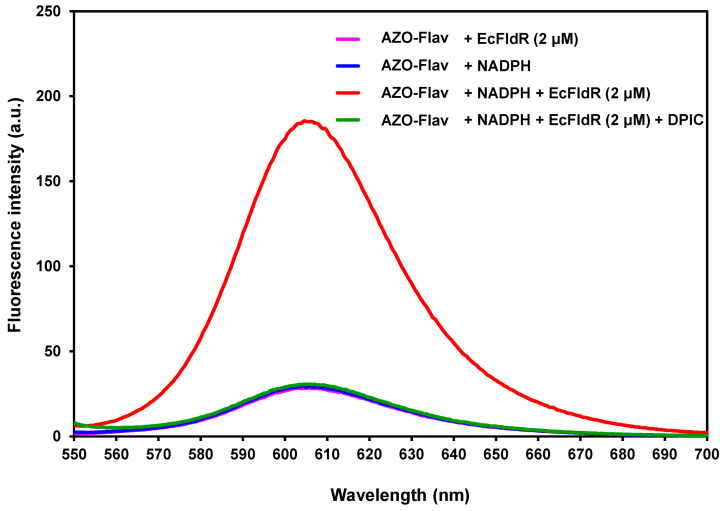
The fluorescent response of **AZO-Flav** (10 μM) in the presence of NADPH (50 μM) catalyzed by *Ec*FldR in 100 mM hypoxic phosphate buffer (pH 7.4) with and without DPIC (50 μM). The spectra were measured at the excitation wavelength of 540 nm.

To investigate the ability of the probe **AZO-Flav** to detect hypoxia, the fluorescence response of **AZO-Flav** towards reductases was tested in vitro (Figure 3). The flavodoxin reductase, *E. coli* FldR (*Ec*FldR), was chosen because it can be simply overexpressed and purified in a large quantity in the lab (Figure S3). Moreover, *Ec*FldR has also been applied to reduce various cytochrome P450 enzymes, including microsomal cytochrome P450, via an electron transfer process [33,34,35]. Therefore, *Ec*FldR could be used to mimic cytochrome P450 reductases in cancer cells. A hypoxic environment was created by purging nitrogen gas for 30 min before adding *Ec*FldR (2 μM) and its cofactor NADPH (50 μM). Subsequently, the mixture was preincubated at 37 °C for 5 min to activate the enzyme prior to the addition of **AZO-Flav** (10 μM). Upon the addition of **AZO-Flav**, a dramatic fluorescent enhancement (Figure 3) was detected; its fluorescent spectrum is similar to that of **Flav-NH_2_**. As proposed in Scheme 1, it suggested that the azo bond was cleaved followed by fragmentation to generate **Flav-NH_2_**. To further confirm the product identity, the reaction mixture was analyzed by HPLC with the standard comigration (Figure S4). In Figure 3, the control reaction (no *Ec*FldR, blue line) did not show fluorescence enhancement, suggesting that **Flav-NH_2_** was resulted from the *Ec*FldR-catalyzed reaction. Furthermore, to confirm that *Ec*FldR catalyzes reduction via electron transfer, which is similar to cytochrome P450 reductases, an experiment for inhibition of the reduction process was performed. Therefore, diphenyliodonium chloride (DPIC), known as an electron scavenger in the electron transport process [36,37,38], was applied to this study. DPIC was added to the mixture prior to the addition of **AZO-Flav**, and its inhibitory effect on the *Ec*FldR activity was investigated. We found that the addition of DPIC (50 μM) in the full reaction led to a very weak fluorescence signal (Figure 3, green line) similar to the ones of the negative control reactions. This suggested that the electron transfer reduction (Scheme 1) was inhibited. In addition, the coenzyme NADPH was also proved to be a key factor in the *Ec*FldR-catalyzed reduction (Figure 3, magenta line).

**Figure 4:**
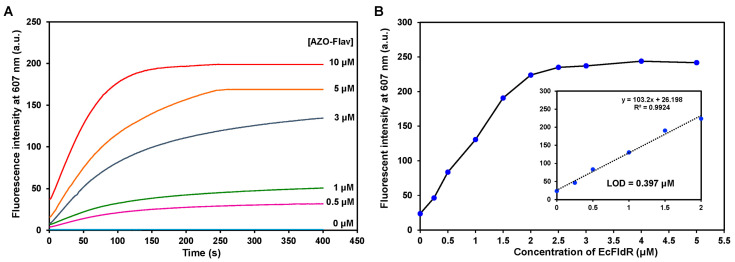
Response of **AZO-Flav** towards *Ec*FldR under simulated hypoxic conditions. (**A**) 400 s time course measurement (λ_ex_ = 540 nm and λ_em_ = 607 nm) of 2 μM of *Ec*FldR and 50 μM of NADPH incubated with **AZO-Flav** at various concentrations (0–10 μM). (**B**) **AZO-Flav** (10 μM) responses to various concentrations of *Ec*FldR (0.5–5.0 μM) in the presence of NADPH (50 μM).

To mimic sensing of cytochrome P450 reductases in cancer cells, linear fluorescence responses with varied concentrations of **AZO-Flav** or *Ec*FldR were investigated to monitor the release of **Flav-NH_2_** from **AZO-Flav** triggered by *Ec*FldR in the presence of excess NADPH by fluorescence spectroscopy (Figure 4). In Figure 4A, the results showed that the fluorescence intensities increased along with **AZO-Flav** concentration in the presence of a fixed concentration of *Ec*FldR and excess NADPH. Furthermore, the fluorescence signals reached the maximum after 2 min, which provides the basis for rapid response detection. Moreover, in all experiments, the fluorescence signals of the reduction product (**Flav-NH_2_**) were higher than the background signals from **AZO-Flav** at 0 min. To determine the limit of detection (LOD) for *Ec*FldR, 10 μM of **AZO-Flav** and 50 μM of NADPH were incubated with varied concentrations of *Ec*FldR (0–5 μM) for 2 min and analyzed by fluorescence spectroscopy (Figure 4B). The results showed that the fluorescence intensities linearly increased along with *Ec*FldR concentration (0–2 μM). Therefore, the LOD value was calculated to be 0.4 μM. 

### 2.5. Specificity of **AZO-Flav** Reduction

**Figure 5:**
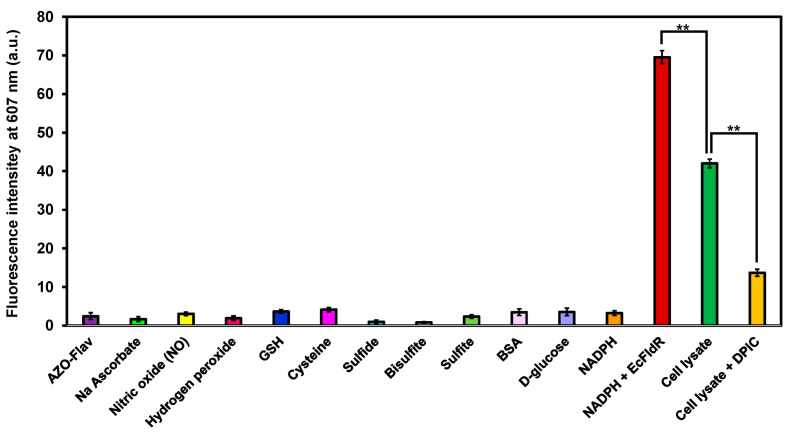
Selectivity of **AZO-flav** (10 μM) towards *Ec*FldR incubated in 100 mM hypoxic phosphate buffer (pH 7.4) containing NADPH (50 μM) and either Na ascorbate (1 mM), nitric oxide (100 μM), hydrogen peroxide (100 μM), GSH (10 mM), cysteine (1 mM), sulfide (1 mM), bisulfite (1 mM), sulfite (1 mM), BSA (1 mg mL^−1^), *D*-glucose (1 mM), NADPH (100 μM), HepG2 cell lysate extract, or HepG2 cell lysate extract with DPIC compared with **AZO-Flav** (10 μM) alone without enzyme. All samples were incubated for 5 min at 37 °C before measuring fluorescent spectra (excitation wavelength = 540 nm). Statistical analysis is based on T-test (** *p* < 0.01).

Because cells contain various metabolites and proteins, we investigated whether they react with **AZO-Flav** to generate false-positive fluorescence signals. **AZO-Flav** was treated with various reductants (sulfide, sulfite, bisulfite, sodium ascorbate, glutathione, and NADPH), biothiol (cysteine), oxidative species (nitric oxide and hydrogen peroxide), bovine serum albumin (BSA), or glucose. As displayed in Figure 5, all substances in very high concentrations did not induce any noticeable fluorescence enhancement compared with the full reaction (NADPH+*Ec*FldR). Moreover, to be applicable for live cell imaging, **AZO-Flav** was also tested in the cell lysate extract. Interestingly, there was a turn-on signal in the cell lysate extract experiment. By adding the electron scavenger, DPIC, we could further confirm that the turn-on signal was majorly from the reduction catalyzed by the reductases inside the cells (Figure 5). To be certain that **AZO-Flav** could be a great candidate for hypoxia detection in tumor environment with high specificity, the following cell assays were performed. 

### 2.6. Hypoxic Cell Imaging

As all above findings support the potential of **AZO-Flav** in hypoxia detection, the probe, **AZO-Flav**, was then applied to monitor the hypoxic condition in human liver carcinoma cells, HepG2. Prior to performing cellular sensing experiments, cytotoxicities of the azo probe, **AZO-Flav**, and its reduction product, **Flav-NH_2_**, were inspected. Cell viability assays using MTT reagent were conducted to determine their safe doses for the live cell detection experiments. The results showed that the cells maintained full viability at concentrations up to 20 μM for both **AZO-Flav** and **Flav-NH_2_** (Figure S5). At higher concentrations (30–50 μM), cell viability decreased to about 65%. Therefore, the optimal concentration ranges for monitoring hypoxia in cells would be 2.5 to 20 μM. 

**Figure 6:**
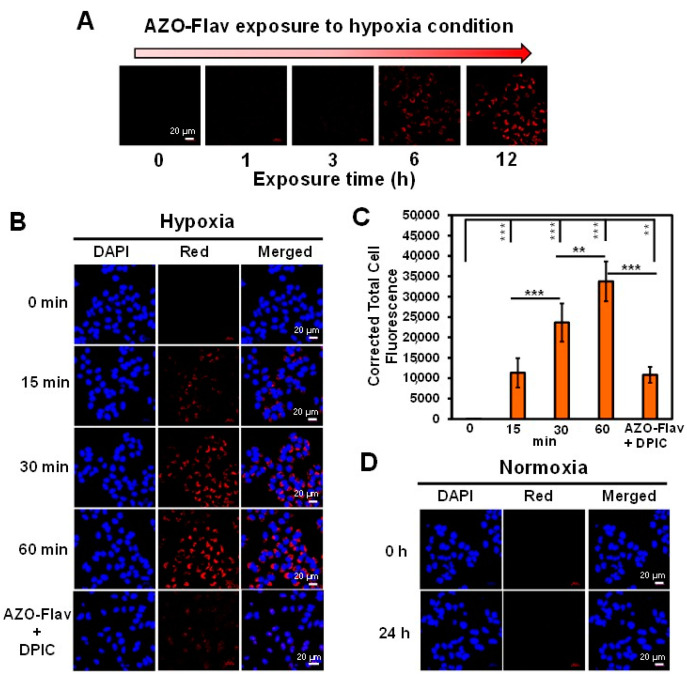
Confocal images of HepG2 cells. (**A**) Different exposure times (0, 1, 3, 6, and 12 h) of HepG2 cells in hypoxic conditions. (**B**) Time dependent hypoxia detection, with hypoxic cells incubated with 5 μM of **AZO-Flav** for 0, 15, 30, and 60 min. DPIC (50 μM) was added after 60 min incubation to inhibit electron transfer. (**C**) Quantitative corrected total cell fluorescence data of images in B were quantified using ImageJ and represent the mean ± SD (n = 100 from three independent experiments). (**D**) The cells incubated with 5 μM of **AZO-Flav** for 0 and 24 h under normoxia. Statistical analysis: One-way ANOVA followed by Tukey’s post-hoc analysis was used for comparison between multiple groups using R studio. P values of less than 0.05 are considered significant (** *p* < 0.01, *** *p* < 0.001).

Consequently, **AZO-Flav** was tested in detection of hypoxia in living cells. The HepG2 cells were incubated in a hypoxia incubator chamber (5% pO_2_) for different duration times to detect graded hypoxic conditions. It was found that the fluorescent signal of the reduction product was clearly observed after the cells were exposed to the low oxygen condition for 6 h and reached the maximum after 12 h incubation (Figure 6A). Therefore, we chose to expose the cells to hypoxia for 12 h for the following experiments. 

After incubation in a hypoxia incubator chamber for 12 h, the cells were treated with **AZO-Flav** for different time durations (15, 30, and 60 min). The fluorescence signal of the reduction product (**Flav-NH_2_**) was found to notably increase over time compared to the signal observed from the cells in normoxic conditions (Figure 6B,C, and the Supporting Video). Moreover, the fluorescence from the enzymatic reaction in hypoxia is comparable with the signal observed from the cells incubated with **Flav-NH_2_** at the same periods (Figure S5). These confirmed that the detected fluorescence signal appeared in a time-dependent manner. In addition, when hypoxic enzyme activity was inhibited by DPIC [7], the fluorescence signal from the cells in the hypoxic environment was suppressed (Figure 6B,C). In contrast, there is no red fluorescent signal observed in normoxic cells, even after incubating with the probe for 24 h (Figure 6D). To observe if photobleaching occurs after hypoxic cells were incubated with **AZO-Flav** for 60 min where the maximal signal is achieved, a video of live cell imaging from 60–120 min was recorded to see if the fluorescence remains stable over time. We found that the signal slightly increased over time, and after 90 min the signal decreased. This implied that the fluorescence from the reduction product is stable for up to 90 min in hypoxic cells (see Supporting Video). Therefore, **AZO-Flav** was shown to be a highly specific probe for hypoxia detection in living cells.

**Figure 7:**
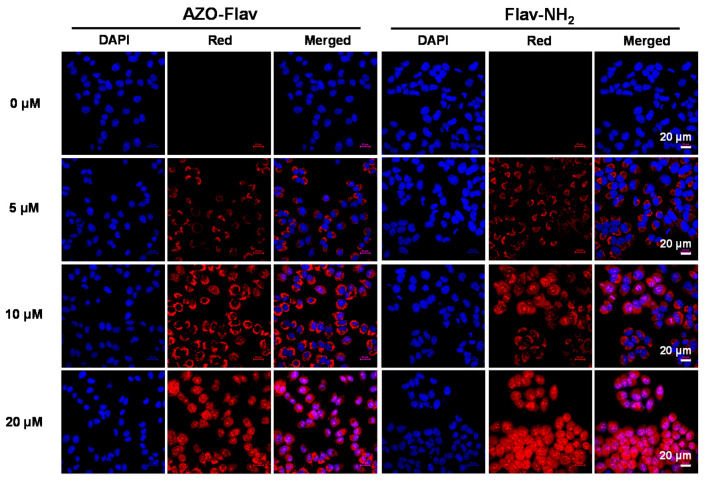
Dose-dependent cellular uptake of **AZO-Flav** and **Flav-NH_2_** at different concentrations (0, 5, 10, and 20 μM) incubated for 60 min under hypoxia.

Dose-dependent internalizations of **AZO-Flav** and **Flav-NH_2_** were also investigated for comparison. As shown in Figure 7, when greater concentrations of **AZO-Flav** and **Flav-NH_2_** were used, the fluorescence signals also increased significantly under hypoxic conditions. Interestingly, at higher concentrations (10–20 μM), the detected signal of **Flav-NH_2_** was found to be localized in the cell nuclei (Hoechst 33342 signal in blue, DAPI channel). This is in good agreement with previous literature reports regarding the observed interaction of flavylium cations with double-stranded DNA and RNA [39,40]. Moreover, at lower concentrations (≤10 μM), the reduction product was found to be localized in some organelles such as lysosomes, Golgi apparatus, and mitochondria with Pearson’s coefficients of 0.60, 0.62, and 0.55, respectively (Figure S7).

Finally, to compare **AZO-Flav** with commercially available hypoxia detection probes such as EF5 [41] and BioTracker 520 Green Hypoxia Dye [22], we list their comparison in Table S1. The key advantages of **AZO-Flav** are (i) convenient synthesis with less steps and (ii) longer emission wavelength which could avoid signals from cell auto-fluorescence. In addition, **AZO-Flav**, which is an activity-based sensor probe, shows superior advantages, including higher sensitivity, ease of synthesis, and improved selectivity, when compared to some protein-based sensors [42] (Table S2). Moreover, our **AZO-Flav** showed the fastest detection of reductase activity among other azo-based fluorescent sensors [1,7,8,9,22,24,43,44,45,46,47] (Table S3). Thus, all experiments ssupport the generation of strongly emissive **Flav-NH_2_** when **AZO-Flav** was in the hypoxic environment, confirming its ability of hypoxia detection in cancer cells.

## 3. Materials and Methods

### 3.1. Instruments and Chemicals

For all reactions, glassware was oven-dried prior to use. All reagents were purchased from commercial sources (TCI, Carlo Erba, and Sigma-Aldrich, Milan, Italy) and used without any further purification. Column chromatography purification was performed on a silica gel (Merck, Germany) as a stationary phase. Analytical thin layer chromatography (TLC) was performed on TLC Silica gel 60 F254 (Merck, Germany) and visualized in a UV cabinet (254 and 365 nm). ^1^H and ^13^C-NMR spectra were recorded on a Bruker-500 MHz spectrometer at room temperature. Chemical shifts of ^1^H-NMR spectra were reported in ppm and calibrated from the residual non-deuterated solvent DMSO-*d_6_* (2.50 ppm). ^1^H-NMR data are reported as the following: chemical shift, multiplicity (s = singlet, d = doublet, t = triplet, q = quartet, m = multiplet), coupling constants, and number of protons. ^13^C-NMR spectra were also recorded in ppm, DMSO-*d_6_* (39.50 ppm). Mass spectra (MS) were measured under high resolution ESI conditions.

### 3.2. Synthesis of **AZO-Flav** and **Flav-NH_2_**

The detailed syntheses of **AZO-Flav** and **Flav-NH_2_** are reported in the Supporting Information.

### 3.3. Spectroscopic Materials and Methods

All UV/vis absorption and fluorescence spectra were recorded on a UV-vis spectrophotometer (T80+ UV/vis spectrometer, PG Instruments Ltd., Lutterworth, UK) and a spectrofluorometer (JASCO FP-8300), respectively, and performed in a quartz cell with 1 cm path length. In all experiments, the stock solutions (1 mM) of **AZO-Flav** and **Flav-NH_2_** were prepared in DMSO. Hypoxic phosphate buffer (100 mM, pH 7.4) was prepared by N_2_ purge for 30 min before measurement. The fluorescence spectra of **AZO-Flav** and **Flav-NH_2_** (10 μM) in 100 mM hypoxic phosphate buffer were recorded with λ_ex_ = 540 nm. 

#### The Study of pH Effect

The fluorescence response of **AZO-Flav** and **Flav-NH_2_** (10 μM) toward different pH values was performed in 100 mM buffer at different pH values (pH = 3, 4, 5, 6, 7, 8, 9, 10, and 11) and measured at λ_ex_ = 540 nm. 

### 3.4. EcFld Reductase Assay

#### 3.4.1. Overexpression and Purification of *Escherichia coli* Flavodoxin Reductase (*Ec*FldR)

##### Plasmid Construction of pET30-*Ec*FldR

The gene encoding *Ec*FldR was amplified from *E. coli* MG1655 genomic DNA by Q5 high-fidelity DNA polymerase (New England Biolabs). The plasmid of pET30-*Ec*FldR was constructed by Gibson assembly of PCR products. The amino acid sequence of overexpressed *Ec*FldR contains a N-terminal His-tag and *Ec*FldR (underlined label). 

MSSHHHHHHSSGENLYFQGGGMADWVTGKVTKVQNWTDALFSLTVHAPVLPFTAGQFTKLGLEIDGERVQRAYSYVNSPDNPDLEFYLVTVPDGKLSPRLAALKPGDEVQVVSEAAGFFVLDEVPHCETLWMLATGTAIGPYLSILQLGKDLDRFKNLVLVHAARYAADLSYLPLMQELEKRYEGKLRIQTVVSRETAAGSLTGRIPALIESGELESTIGLPMNKETSHVMLCGNPQMVRDTQQLLKETRQMTKHLRRRPGHMTAEHYW.

##### Overexpression and Purification of *Ec*FldR

Ten milliliters of overnight culture of *E. coli* BL21(DE3) containing pET30-*Ec*FldR was inoculated into 1 L of Luria-Bertani broth (LB) with 50 µg/mL kanamycin. The culture was shaken at 200 rpm and 37 °C until OD_600_ reached about 0.6. Protein expression was induced by addition of isopropyl-β-*D*-1-thiogalactopyranoside (IPTG) with a final concentration of 200 µM. The culture mixture was shaken for an additional 16 h at 200 rpm and 20 °C. Subsequently, cells were collected by centrifugation (5000 rpm, 25 min, 8 °C) and kept at −80 °C till purification. The harvested cells were thawed and resuspended in the lysis buffer (300 mM NaCl, 50 mM NaH_2_PO_4_, and 10 mM imidazole). The cells were lysed by sonication (1.5 s cycle, 50% duty) on ice, followed by centrifugation at 12,000 rpm and 4 °C for 40 min. The supernatant was loaded onto a Ni-NTA column (QIAGEN) and the proteins were eluted by the manufacturer’s instructions. After elution, the pure fractions were combined and concentrated, followed by incubation with 1 mM of flavin adenine dinucleotide (FAD). The unbound FAD was removed by a 10DG column (BioRad) pre-equilibrated with 100 mM Tris-HCl, 30% glycerol, pH 7.5. The purified protein was aliquoted and stored at −80 °C. The SDS-PAGE analysis is showed in Figure S2.

#### 3.4.2. Response towards *Ec*Fld Reductase

2 μM of *Ec*FldR and 50 μM of NADPH were preincubated at 37 °C for 5 min in 100 mM hypoxic phosphate buffer (pH 7.4). The reaction was initiated by the addition of 10 μM of **AZO-Flav** and analyzed by fluorescence spectrometry at λ_ex_ = 540 nm and λ_em_ = 607 nm.

#### 3.4.3. Selectivity towards *Ec*Fld Reductase

All interference stocks (sodium ascorbate, nitric oxide (NO), hydrogen peroxide (H_2_O_2_), glutathione (GSH), cysteine, Na_2_S (H_2_S), Na_2_O_5_S_2_ (HSO_3_^−^), Na_2_SO_3_, bovine serum albumin (BSA), *D*-glucose, and NADPH) were prepared in 100 mM hypoxic phosphate buffer (pH 7.4). 10 μM of **AZO-Flav** was added into hypoxic phosphate buffer containing 50 μM of NADPH and each interference.

For lysate preparation, HepG2 cells cultured in complete media (See Section 3.5.1) on a T-25 flask were washed twice with cold PBS. Subsequently, RIPA buffer (Thermo Scientific, Waltham, MA, USA) was added to the cells and the flask was kept on ice for 5 min, swirling occasionally. The cells were removed from the flask using a cell scraper, then transferred into a microcentrifuge tube. Collected cells were then centrifuged at ~14,000× *g* for 15 min. The supernatant was transferred to a new tube for further analysis. 

For fluorescence experiments, all samples were incubated at 37 °C for 5 min before adding **AZO-Flav** (10 μM). The emission spectra were recorded at λ_ex_ = 540 nm and λ_em_ = 607 nm. 

#### 3.4.4. Limit of Detection (LOD) of **AZO-Flav** Reduction toward *Ec*FldR

100 mM hypoxic phosphate buffer containing various concentrations of *Ec*FldR (0, 0.25, 0.5, 1, 1.5, 2, 3, 4, and 5 μM) and 50 μM of NADPH was preincubated at 37 °C for 5 min. After that, **AZO-Flav** was added to the solution at a final concentration of 10 μM. The fluorescence intensity of the reduction product, **Flav-NH_2_**, was analyzed by a fluorescence spectrophotometer (λ_ex_ = 540 nm and λ_em_ = 607 nm).

#### 3.4.5. HPLC for **AZO-Flav** with *Ec*FldR

The reaction solutions were prepared in 100 mM hypoxic phosphate buffer containing 2 μM of *Ec*FldR and 50 μM of NADPH; these were preincubated at 37 °C for 5 min. **AZO-Flav** was then added to the final concentration of 10 μM to initiate the reaction. The mixture was incubated for 5 min. The reaction was quenched by adding 50% acetonitrile to precipitate *Ec*FldR followed by centrifugation at 10,000 rpm for 5 min to remove the protein. The supernatant was analyzed by HPLC. Reverse phase HPLC analysis was performed on an Agilent HPLC 1100 using a column of ZORBAX Eclipse XDB-C18 (4.6 mm × 150 mm, 5 µm ID). The solvents were solvent A (water + 0.1% TFA) and solvent B (acentonitrile + 0.1% TFA). The linear gradient was as follows: 0 min: 100% A; 2 min: 95% A, 5% B; 5 min: 85% A, 15% B; 10 min: 5% A, 95% B; 12 min: 5% A, 95% B; 14 min: 95% A, 5% B; 16 min: 100% A; 20 min: 100% A. The flow rate was 1 mL/min. The analysis was monitored by a UV-Vis detector at a wavelength of 560 nm.

### 3.5. Cell Culture and Confocal Imaging

#### 3.5.1. Cell Culture

HepG2 (a human liver cancer cell line, purchased from ATCC) cells were cultured on a 75 cm^3^ culture flask in a complete medium, Dulbecco’s Modified Eagle’s Media (DMEM, Hyclone) supplemented with 10% fetal bovine serum (FBS, Gibco) and 1% penicillin–streptomycin (Corning). The cells were incubated at 37 °C in a humidified atmosphere containing 5% CO_2_. Under hypoxia conditions, the cells were incubated in a hypoxia incubator chamber (STEMCELL Technologies Inc., Vancouver, BC, Canada).

#### 3.5.2. Cell Imaging

HepG2 cells were seeded on an 8-well chambered coverglass (LabTek, Nunc) at 1 × 10^4^ per well and incubated at 37 °C for 24 h. For *time-dependent cellular uptake*, the cells were incubated under normoxic (humidified 95% air, 5% CO_2_ atmosphere) and hypoxic (5% pO_2_) conditions at 37 °C for 12 h. Subsequently, the cells were treated with 5 μM of **AZO-Flav** (or **Flav-NH_2_**) in FBS-free DMEM for 0, 15, 30, and 60 min. For *dose dependent cellular uptake*, the cells were treated with 0, 5, 10, and 20 μM of **AZO-Flav** and **Flav-NH_2_** in FBS-free DMEM for 60 min. After the incubation, the cells were washed with PBS buffer (0.01 M, pH 7.4) three times and treated with fresh media containing 1.0 µM of Hoechst 33342 (Thermo Fisher Scientific) for 10 min before being imaged by a Laser Scanning Confocal Microscope (LSCM, Nikon A1Rsi). Laser sources were as follows: excitation: 561 nm and emission: 595 nm/50 nm (for **AZO-Flav** or **Flav-NH_2_**), and excitation: 405 nm and emission: 450 nm/50 nm (for Hoechst 33342) using a 60X oil immersion objective lens. Quantitative corrected total cell fluorescence data were quantified using ImageJ and represented the mean ± SD (100 cells from three independent experiments, *n* = 3).

#### 3.5.3. Hypoxia Inhibitory Effect

HepG2 cells were seeded on an 8-well chambered coverglass (LabTek, Nunc) at 1 × 10^4^ per well and incubated at 37 °C for 24 h. Subsequently, the cells were treated with 0, 100, and 200 μM of diphenyliodonium chloride (DPIC, TCI) and incubated under hypoxic (5% pO_2_) conditions at 37 °C for 12 h. After incubation, the cells were treated with 5 μM of **AZO-Flav** in FBS-free DMEM for 60 min. Then, the cells were washed with PBS buffer (0.01 M, pH 7.4), stained with Hoechst 33342, and visualized under LSCM. 

#### 3.5.4. Cell Viability Assay of AZO-Flav and Flav-NH_2_

HepG2 cells were seeded on a 96-well cell culture plate at approximately 7 × 10^3^ cells per well and incubated for 24 h. Cells were then treated with different concentrations of **AZO-Flav** and **Flav-NH_2_** (0, 2.5, 5, 10, 20, 30, 40, and 50 μM) for 24 h. After incubation, the cells were washed with PBS (three times) before adding 25 μL (0.5 mg mL^−1^) of MTT reagent (methylthiazolyldiphenyltetrazolium bromide, Sigma-Aldrich) in 0.01 M PBS (pH 7.4) for 3 h. After supernatant removal, DMSO (100 μL) was added to dissolve the formazan product which was detected at a wavelength of 560 nm using a microplate reader (BMG Labtech/SPECTROstar Nano).

## 4. Conclusions

**AZO-Flav** was successfully developed as a hypoxia-responsive probe. In an enzyme-catalyzed reduction, **AZO-Flav** exhibited high selectivity and sensitivity towards an electron transfer process in the presence of the reductase and its cofactor, NADPH, with a limit of detection about 0.4 μM. The azo bond was cleaved via the enzymatic reaction to release the corresponding amine (**Flav-NH_2_**) that provided the strong fluorescence turn-on signal. The capability of **AZO-Flav** to detect hypoxia in cancer cells was confirmed by cell imaging experiments. Fluorescence intensities were found to increase up to 26-fold when hypoxic cells were incubated with **AZO-Flav** for 60 min. Moreover, fluorescence signals from the hypoxic cells can be suppressed by the inhibition of the electron transfer process, suggesting the azo bond reduction of **AZO-Flav** is associated with reductase in the hypoxic tumor. Lastly, the detected fluorescence signals from the cell nuclei after hypoxic cells incubated with **AZO-Flav** confirmed the existing of the reduction product (**Flav-NH_2_**) inside the cells. Therefore, **AZO-Flav** showed great potential in its application toward in vivo hypoxia detection.

## Data Availability

Data are contained within the article or Supplementary Materials. The data set can be found via doi:10.6084/m9.figshare.15117108.

## Supplementary Materials

The followings are available online. General procedure for the synthesis of **AZO-Flav** and **Flav-NH_2_** and compound characterizations; Figure S1: Calibration curve of **Flav-NH_2_** (λex = 540 nm and λem = 607 nm); Figure S2: pH effect in 100 mM phosphate buffer at pH = 3, 4, 5, 6, 7, 8, 9, 10, and 11 with λex = 540 nm and λem = 607 nm. (a) 10 μM of **Flav-NH_2_** and (b) 10 μM of **AZO-Flav**; Figure S3: SDS-PAGE analysis of purified *Ec*FldR; Figure S4: HPLC analysis of the metabolism of **AZO-Flav** when reacted with *Ec*FldR reductase. **AZO-Flav** (10 µM) and NADPH (50 μM) were treated with *Ec*FldR reductase (2 μM) for 5 min. HPLC profiles were detected by UV/Vis at 560 nm; Figure S5: MTT assay of **AZO-flav** and **Flav-NH_2_** in HepG2 at different concentrations, incubated for 24 h; Figure S6: Time-dependent cellular uptake of **Flav-NH_2_** incubated for 0, 15, 30, and 60 min; Figure S7: Confocal images of **AZO-Flav** incubated with hypoxic HepG2 cells and colocalized with sub-organelle trackers. Table S1: Comparison of **AZO-Flav** with commercially available hypoxia detection probes; Table S2: Comparison of **AZO-Flav** with a reported protein-based sensor; Table S3: The structures and O2 responses of azo-based probes. Video files of imaging of cells (hypoxia and normoxia) incubated with **AZO-Flav** from 15–60 min; video recording of photobleaching behavior of the dye in hypoxic cells from 60–120 min.

## Sample Availability

Samples of **AZO-Flav** and **Flav-NH_2_** are available from the authors.

## References

[1] Luo S., Liu Y., Wang F., Fei Q., Shi B., An J., Zhao C., Tung C.-H. (2016). A fluorescent turn-on probe for visualizing lysosomes in hypoxic tumor cells. Analyst.

[2] Wheeler K.T., Wang L.-M., A Wallen C., Childers S.R., Cline J.M., Keng P.C., Mach R.H. (2000). Sigma-2 receptors as a biomarker of proliferation in solid tumours. Br. J. Cancer.

[3] Höckel M., Vaupel P. (2001). Tumor Hypoxia: Definitions and Current Clinical, Biologic, and Molecular Aspects. J. Natl. Cancer Inst..

[4] Al Tameemi W., Dale T.P., Al-Jumaily R.M.K., Forsyth N.R. (2019). Hypoxia-Modified Cancer Cell Metabolism. Front. Cell Dev. Biol..

[5] Dutta B., Yan R., Lim S.K., Tam J.P., Sze S.K. (2014). Quantitative Profiling of Chromatome Dynamics Reveals a Novel Role for HP1BP3 in Hypoxia-induced Oncogenesis. Mol. Cell. Proteom..

[6] Rademakers S.E., Span P., Kaanders J.H., Sweep F., Van Der Kogel A.J., Bussink J. (2008). Molecular aspects of tumour hypoxia. Mol. Oncol..

[7] Wang C., Zhang S., Huang J., Cui L., Hu J., Tan S. (2019). Novel designed azo substituted semi-cyanine fluorescent probe for cytochrome P450 reductase detection and hypoxia imaging in cancer cells. RSC Adv..

[8] Cai Q., Yu T., Zhu W., Xu Y., Qian X. (2015). A turn-on fluorescent probe for tumor hypoxia imaging in living cells. Chem. Commun..

[9] Kiyose K., Hanaoka K., Oushiki D., Nakamura T., Kajimura M., Suematsu M., Nishimatsu H., Yamane T., Terai T., Hirata Y. (2010). Hypoxia-Sensitive Fluorescent Probes for in Vivo Real-Time Fluorescence Imaging of Acute Ischemia. J. Am. Chem. Soc..

[10] Luo S., Zou R., Wu J., Landry M.P. (2017). A Probe for the Detection of Hypoxic Cancer Cells. ACS Sensors.

[11] Kumari R., Sunil D., Ningthoujam R.S., Kumar N.A. (2019). Azodyes as markers for tumor hypoxia imaging and therapy: An up-to-date review. Chem. Interactions.

[12] Fradette C., Du Souich P. (2004). Effect of hypoxia on cytochrome P450 activity and expression. Curr. Drug Metab..

[13] Patterson A., Saunders M.P., Chinje E.C., Talbot D.C., Harris A., Strafford I.J. (1997). Overexpression of human NADPH:cytochrome c (P450) reductase confers enhanced sensitivity to both tirapazamine (SR 4233) and RSU 1069. Br. J. Cancer.

[14] Williams K.J., Cowen R.L., Stratford I.J. (2001). Hypoxia and oxidative stress. Tumour hypoxia--therapeutic considerations. Breast Cancer Res..

[15] Wright A., Song J., Cravatt B.F. (2009). A Suite of Activity-Based Probes for Human Cytochrome P450 Enzymes. J. Am. Chem. Soc..

[16] Huttunen K.M., Mähönen N., Raunio H., Rautio J. (2008). Cytochrome P450-activated prodrugs: Targeted drug delivery. Curr. Med. Chem..

[17] Rodriguez-Antona C., Ingelmansundberg M. (2006). Cytochrome P450 pharmacogenetics and cancer. Oncogene.

[18] Scripture C.D., Sparreboom A., Figg W.D. (2005). Modulation of cytochrome P450 activity: Implications for cancer therapy. Lancet Oncol..

[19] McFadyen M.C.E., Melvin W.T., I Murray G. (2004). Cytochrome P450 enzymes: Novel options for cancer therapeutics. Mol. Cancer Ther..

[20] Wu J., Guan X., Dai Z., He R., Ding X., Yang L., Ge G. (2021). Molecular probes for human cytochrome P450 enzymes: Recent progress and future perspectives. Coord. Chem. Rev..

[21] Feng L., Ning J., Tian X., Wang C., Yu Z., Huo X., Xie T., Zhang B., James T.D., Ma X. (2021). Fluorescent probes for the detection and imaging of Cytochrome P450. Coord. Chem. Rev..

[22] Piao W., Tsuda S., Tanaka Y., Maeda S., Liu F., Takahashi S., Kushida Y., Komatsu T., Ueno T., Terai T. (2013). Development of azo-based fluorescent probes to detect different levels of hypoxia. Angew. Chem. Int. Ed. Engl..

[23] Wilson W.R., Hay M. (2011). Targeting hypoxia in cancer therapy. Nat. Rev. Cancer.

[24] Cui L., Shi Y., Zhang S., Yan L., Zhang H., Tian Z., Gu Y., Guo T., Huang J. (2017). A NIR turn-on fluorescent probe applied in cytochrome P450 reductase detection and hypoxia imaging in tumor cells. Dye. Pigment..

[25] Pina F., Petrov V., Laia C.A.T. (2012). Photochromism of flavylium systems. An overview of a versatile multistate system. Dyes Pigm..

[26] Pina F., Melo M.J., Laia C., Parola A.J., Lima J.C. (2012). Chemistry and applications of flavylium compounds: A handful of colours. Chem. Soc. Rev..

[27] Kim S., Yoon J., Yoon S., Lee M. (2021). Ratiometric Fluorescence Assay for Nitroreductase Activity: Locked-Flavylium Fluorophore as a NTR-Sensitive Molecular Probe. Molecules.

[28] Gong X., Yang X.-F., Zhong Y., Chen H., Li Z. (2016). A flavylium-based turn-on fluorescent probe for imaging hydrogen polysulfides in living cells. RSC Adv..

[29] Bandara H.M., Burdette S.C. (2012). Photoisomerization in different classes of azobenzene. Chem. Soc. Rev..

[30] Chevalier A., Renard P.-Y., Romieu A. (2017). Azo-Based Fluorogenic Probes for Biosensing and Bioimaging: Recent Advances and Upcoming Challenges. Chem. Asian J..

[31] Ren T.-B., Xu W., Jin F., Cheng D., Zhang L., Yuan L., Zhang X. (2017). Rational Engineering of Bioinspired Anthocyanidin Fluorophores with Excellent Two-Photon Properties for Sensing and Imaging. Anal. Chem..

[32] Thews O., Riemann A. (2019). Tumor pH and metastasis: A malignant process beyond hypoxia. Cancer Metastasis Rev..

[33] Jenkins C.M., Waterman M.R. (1998). NADPH-Flavodoxin Reductase and Flavodoxin from Escherichia coli: Characteristics as a Soluble Microsomal P450 Reductase. Biochemistry.

[34] Jenkins C.M., Waterman M.R. (1994). Flavodoxin and NADPH-flavodoxin reductase from Escherichia coli support bovine cytochrome P450c17 hydroxylase activities. J. Biol. Chem..

[35] Jenkins C.M., Waterman M.R. (1999). Flavodoxin As A Model for The P450-Interacting Domain of Nadph Cytochrome P450 Reductase. Drug Metab. Rev..

[36] Begleiter A., Leith M.K., Patel D., Hasinoff B.B. (2007). Role of NADPH cytochrome P450 reductase in activation of RH1. Cancer Chemother. Pharmacol..

[37] Kleniewska P., Piechota-Polanczyk A., Skibska B., Gorąca A. (2012). The NADPH Oxidase Family and its Inhibitors. Arch. Immunol. Ther. Exp..

[38] Panday A., Sahoo M., Osorio D., Batra S. (2015). NADPH oxidases: An overview from structure to innate immunity-associated pathologies. Cell. Mol. Immunol..

[39] Crnolatac I., Giestas L., Horvat G., Parola A.J., Piantanida I. (2020). Flavylium Dye as pH-Tunable Fluorescent and CD Probe for Double-Stranded DNA and RNA. Chemosensors.

[40] Sarma A.D., Sharma R. (1999). Anthocyanin-DNA copigmentation complex: Mutual protection against oxidative damage. Phytochemistry.

[41] Koch C.J. (2002). [1] Measurement of absolute oxygen levels in cells and tissues using oxygen sensors and 2-nitroimidazole EF5. Methods Enzymol..

[42] Iglesias P., Penas C., Barral-Cagiao L., Pazos E., Costoya J.A. (2019). A Bio-inspired Hypoxia Sensor using HIF1a-Oxygen-Dependent Degradation Domain. Sci. Rep..

[43] Uddin I., Evans S.M., Craft J.R., Marnett L.J., Uddin J., Jayagopal A. (2015). Applications of Azo-Based Probes for Imaging Retinal Hypoxia. ACS Med. Chem. Lett..

[44] Tian Y., Li Y., Jiang W.-L., Zhou D.-Y., Fei J., Li C.-Y. (2019). In-Situ Imaging of Azoreductase Activity in the Acute and Chronic Ulcerative Colitis Mice by a Near-Infrared Fluorescent Probe. Anal. Chem..

[45] Zhou Y., Maiti M., Sharma A., Won M., Yu L., Miao L.X., Shin J., Podder A., Bobba K.N., Han J. (2018). Azo-based small molecular hypoxia responsive theranostic for tumor-specific imaging and therapy. J. Control. Release.

[46] Chevalier A., Piao W., Hanaoka K., Nagano T., Renard P.-Y., Romieu A. (2015). Azobenzene-caged sulforhodamine dyes: A novel class of ‘turn-on’ reactive probes for hypoxic tumor cell imaging. Methods Appl. Fluoresc..

[47] Huang J., Wu Y., Zeng F., Wu S. (2019). An Activatable Near-Infrared Chromophore for Multispectral Optoacoustic Imaging of Tumor Hypoxia and for Tumor Inhibition. Theranostics.

