# Supplementary material for: Flavylium-Based Hypoxia-Responsive Probe for Cancer Cell Imaging
Source: Molecules. 2021 Aug 15;26(16):4938. doi: 10.3390/molecules26164938 (PMC8400153; doi:10.3390/molecules26164938)
Supplement: Supplementary file 1 [file molecules-26-04938-s001.zip › Supporting information_proof.pdf]

Flavylium-Based Hypoxia-Responsive Probe for Cancer Cell Imaging

Supporting Information

Thitima Pewklang,[1] Sirawit Wet-osot,[1] Sirilak Wangngae,[1] Uthumporn Ngivprom,[1] Kantapat Chansaenpak,[2] Chuthamat Duangkamol,[1] Rung-Yi Lai*,[1] Parinya Noisa,[3] Mongkol Sukwattanasinitt,[4] Anyanee Kamkaew*,[1]

[1] School of Chemistry, Institute of Science, Suranaree University of Technology, Nakhon Ratchasima 30000, Thailand

[2] National Nanotechnology Center, National Science and Technology Development Agency, Thailand Science Park, Pathum Thani 12120, Thailand

[3] Laboratory of Cell-Based Assays and Innovations, School of Biotechnology, Institute of Agricultural Technology, Suranaree University of Technology, Nakhon Ratchasima 30000, Thailand

[4] Thailand Nanotec-CU Center of Excellence on Food and Agriculture, Department of Chemistry, Faculty of Science, Chulalongkorn University, Bangkok 10330, Thailand

Corresponding authors' email: rylai@sut.ac.th; anyanee@sut.ac.th

1. Experimental Data S2
2. ^{1}H and ^{13}C NMR and Mass spectra S4
3. Spectroscopic and methods S11
4. Enzymatic assay S12
5. Biological studies S13

1. Experimental Data

General procedure for the synthesis of AZO-Flav and Flav-NH$_2$ and compound characterizations.

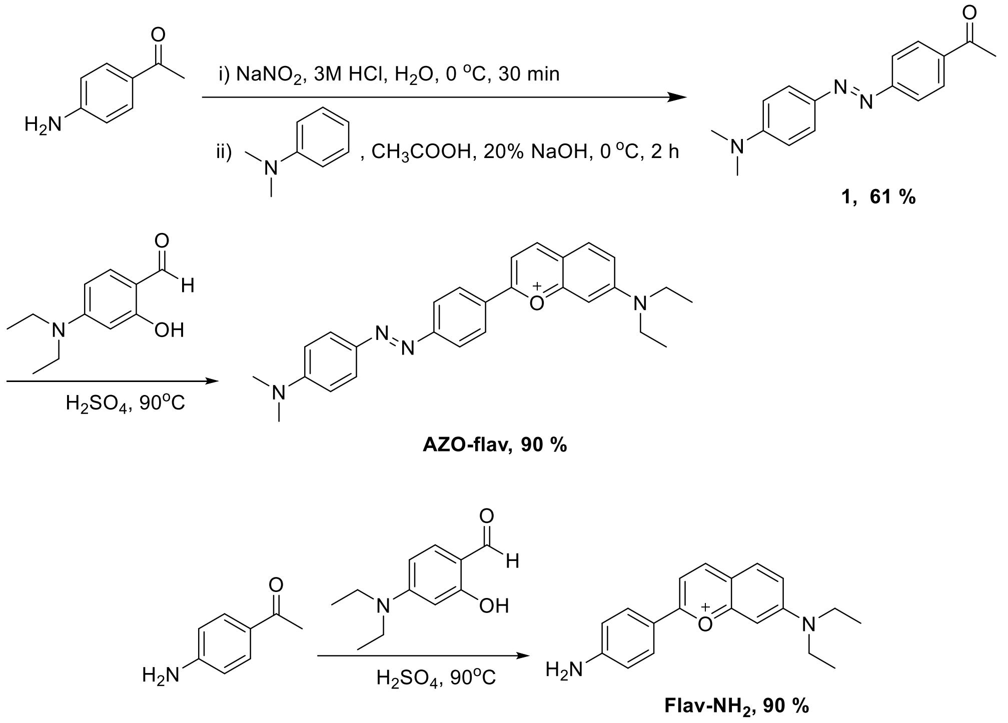

Scheme S1. Syntheses of AZO-Flav and Flav-NH$_2$

(E)-1-(4-((4-(dimethylamino)phenyl)diazenyl)phenyl)ethan-1-one (1)

4-Aminoacetophenone (0.83 g, 5.0 mmol) was dissolved in a 3 M HCl solution (5.0 mL). A solution of sodium nitrite (0.38 g, 5.5 mmol) in distilled water (5.0 mL) was then added dropwise to the solution over 5 min at 0 °C. The mixture was stirred at 0 °C for 30 min before addition slowly of *N,N*-Dimethylphenylamine (0.61 g, 5.0 mmol) in 3 mL acetic acid and cooled to 0 °C. The resultant colored mixture was precipitated by addition of NaOH solution (20%). After the pH reached 7–8, the solution was stirred for a further 2 h at 0 °C. The precipitate was washed with 1:1 ethanol/water and recrystallized with acetone, generating a red solid (0.81 g, 61%). ^{1}H NMR (500 MHz, DMSO-d$_6$): δ = 8.17 (d, *J* = 10.0 Hz, 2H), 7.93 (d, *J* = 10.0 Hz, 2H), 7.91 (d, *J* = 10.0 Hz, 2H), 6.93 (d, *J* = 10.0 Hz, 2H), 3.16 (s, 6H), 2.70 (s, 3H). ^{13}C NMR (125 MHz, DMSO-d$_6$): δ = 197.7, 155.6,153.5, 143.2, 137.2, 130.0, 125.8, 122.3, 112.1, 27.3. HRMS (ESI) calcd for $C_{16}H_{17}N_3NaO$ [M + Na]$^+$ 290.1264, found 290.1265.

(E)-7-(diethylamino)-2-(4-((4-(dimethylamino)phenyl)diazenyl)phenyl) chromenylium (AZO-Flav)

4-(diethylamino)-2-hydroxybenzaldehyde (0.193 g, 1 mmol) and **1** (0.267 g, 1 mmol) was dissolved in 5 ml of concentrated H_2SO_4. After stirred at 90 °C for 2h, the reaction mixture was cooled and poured onto 20 g of ice. Then 1 mL of perchloric acid (70%) was added. The precipitate was filtered and washed with deionized water to afford the crude product, which was purified by silica gel flash column chromatography (5% MeOH/DCM) to give AZO-flav as a dark purple solid (0.382 g, 90%).^{1}H NMR (500 MHz, DMSO-d_6): δ = 8.71 (d, *J* = 8.0 Hz, 1H), 8.36 (d, *J* = 9.0 Hz, 2H), 8.07 (d, *J* = 8.0 Hz, 1H), 7.93 (d, *J* = 9.0 Hz, 1H), 7.86 (d, *J* = 9.0 Hz, 2H), 7.74 (d, *J* = 9.0 Hz, 2H), 7.45 (dd, *J* = 9.5, 2.5 Hz, 1H), 7.30 (s, 1H), 6.76 (d, *J* = 9.0 Hz, 2H), 3.66 (q, *J* = 7.0 Hz, 4H), 2.99 (s, 6H), 1.17 (t, *J* = 6.0 Hz, 6H). ^{13}C NMR (125 MHz, DMSO-d_6): δ = 165.0, 159.7, 156.9, 155.8, 153.9, 149.1, 143.4, 133.0, 129.9, 129.5, 126.3, 123.0, 120.1, 119.4, 112.3, 110.1, 96.4, 46.3, 40.4, 12.8. HRMS (ESI) calcd for $C_{27}H_{29}N_4O$ $[M]^+$ 425.2336, found 425.2336.

2-(4-aminophenyl)-7-(diethylamino)chromenylium (Flav-NH$_2$)

4-(diethylamino)-2-hydroxybenzaldehyde (0.193 g, 1 mmol) and 4-(diethylamino)-2-hydroxybenzaldehyde (0.135 g, 1 mmol) was dissolved in 5 ml of concentrated H_2SO_4. After stirred at 90 °C for 2 h, the reaction mixture was cooled and poured onto 20 g of ice. Then 1 mL of perchloric acid (70%) was added. The precipitate was filtered and washed with deionized water to afford the crude product, which was purified by silica gel flash column chromatography (5% MeOH/DCM) to give Flav-NH_2 as a dark purple solid (0.264 g, 90 %).^{1}H NMR (500 MHz, DMSO-d_6): δ = 8.61 (d, *J* = 8.5 Hz, 1H), 8.24 (d, *J* = 8.5 Hz, 2H), 7.92 (d, *J* = 9.5 Hz, 1H), 7.89 (d, *J* = 8.5 Hz, 1H), 7.35 (dd, *J* = 9.5, 2.0 Hz, 1H), 7.28 (s, 1H), 6.85 (d, *J* = 8.5 Hz, 2H), 3.71 (q, *J* = 7.0 Hz, 4H), 1.30 (t, *J* = 14.0 Hz, 6H). ^{13}C NMR (125 MHz, DMSO-d_6): δ = 168.2, 158.2, 157.1, 154.9, 147.7, 132.2, 131.9, 116.1, 115.7, 115.4, 114.8, 108.2, 96.4, 45.5, 12.9. HRMS (ESI) calcd for $C_{19}H_{21}N_2O$ $[M]^+$ 293.1648, found 293.1647.

2. ¹H, ¹³C, and Mass spectra of AZO-flav and Flav-NH₂

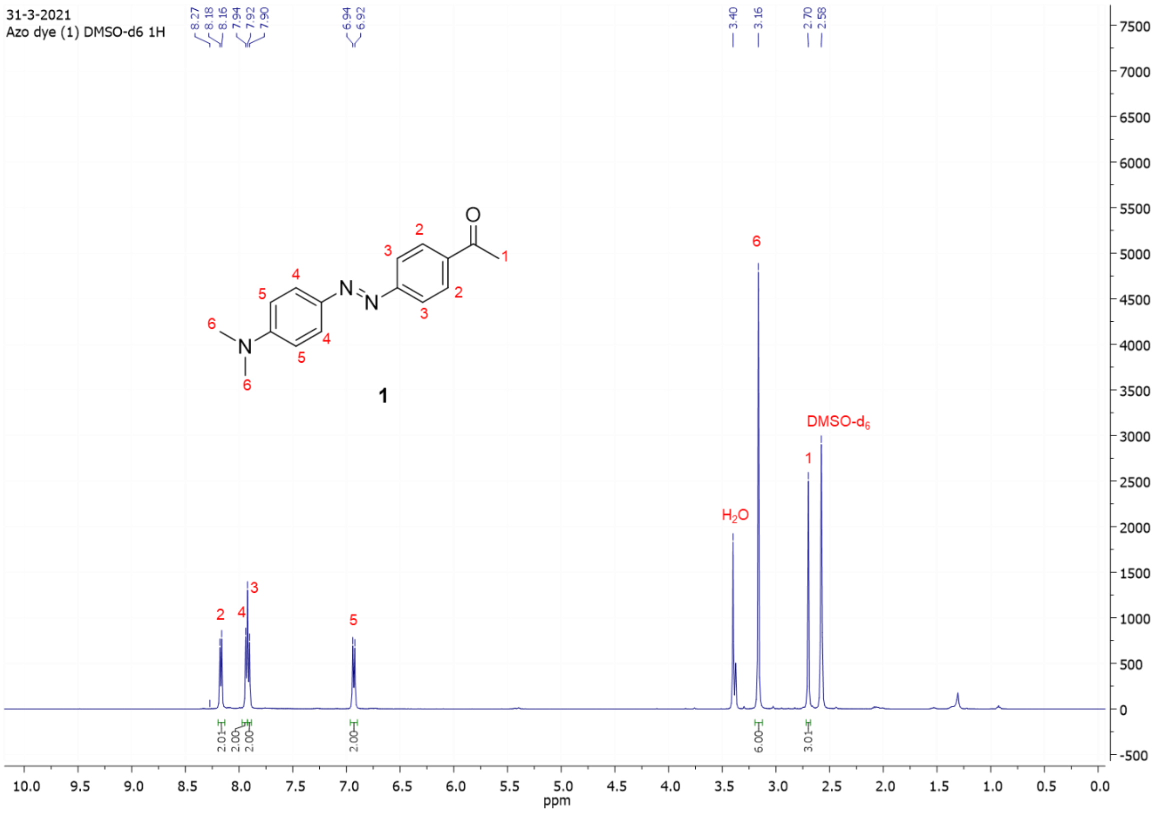

^{1}H NMR spectra of **1** in DMSO-d$_6$

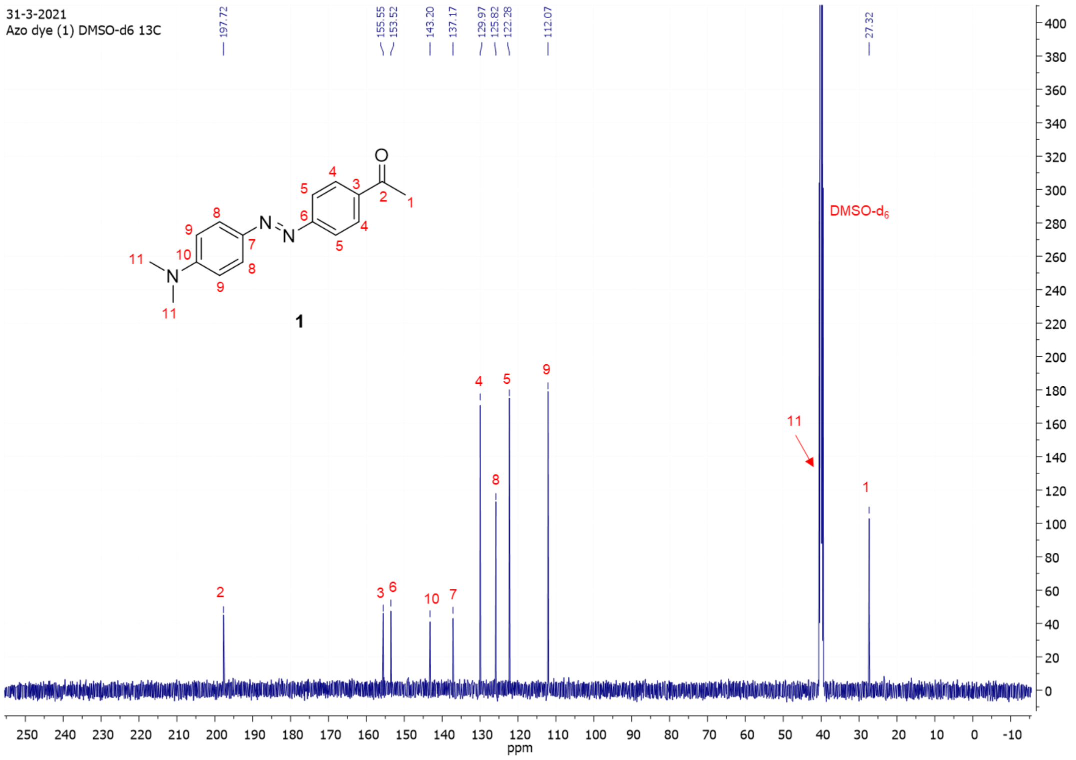

^{13}C NMR spectra of **1** in DMSO-d$_6$

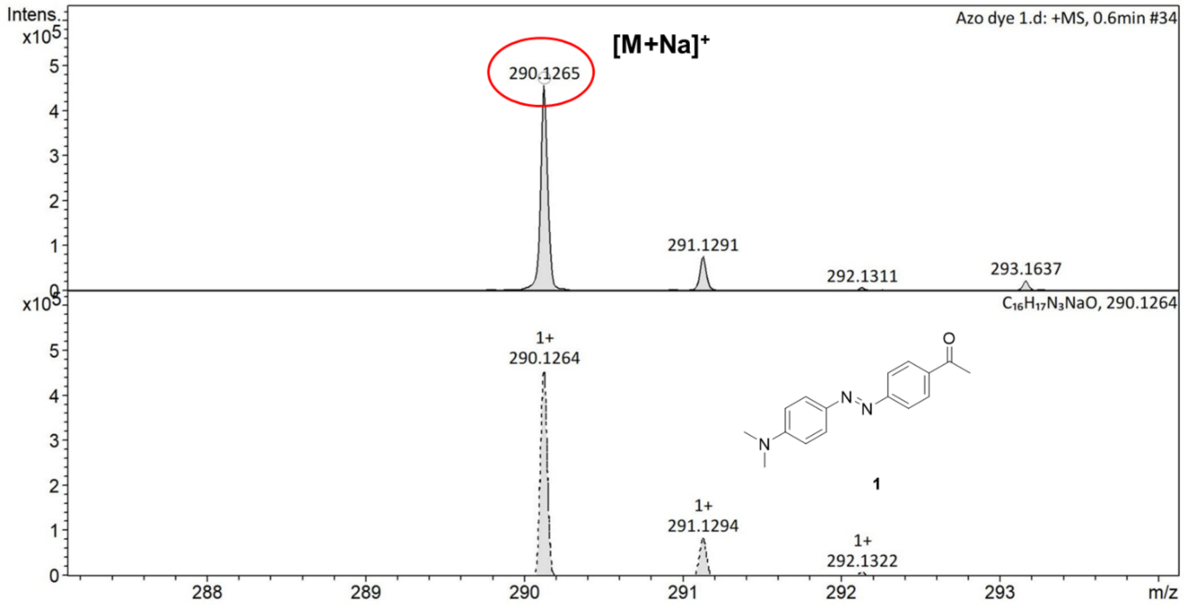

High resolution ESI$^+$ spectra of **1**

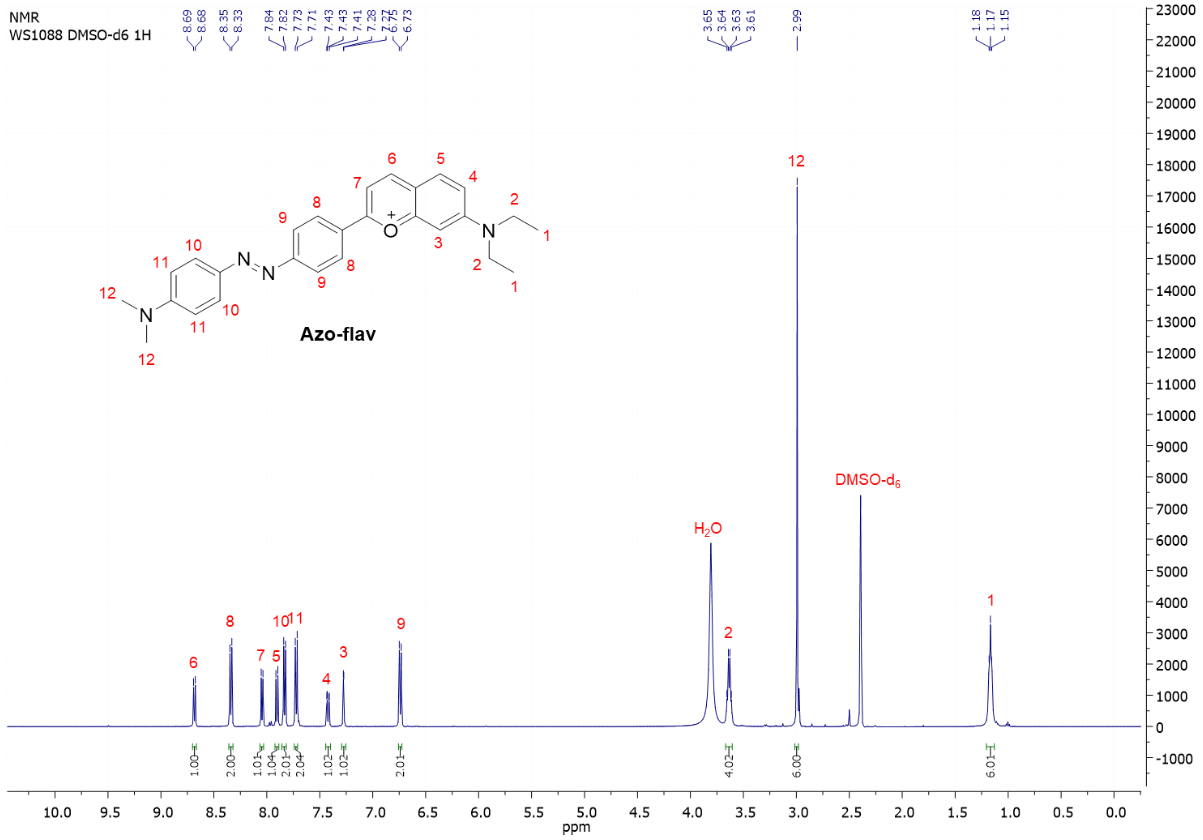

^{1}H NMR spectra of AZO-flav in DMSO-d_6

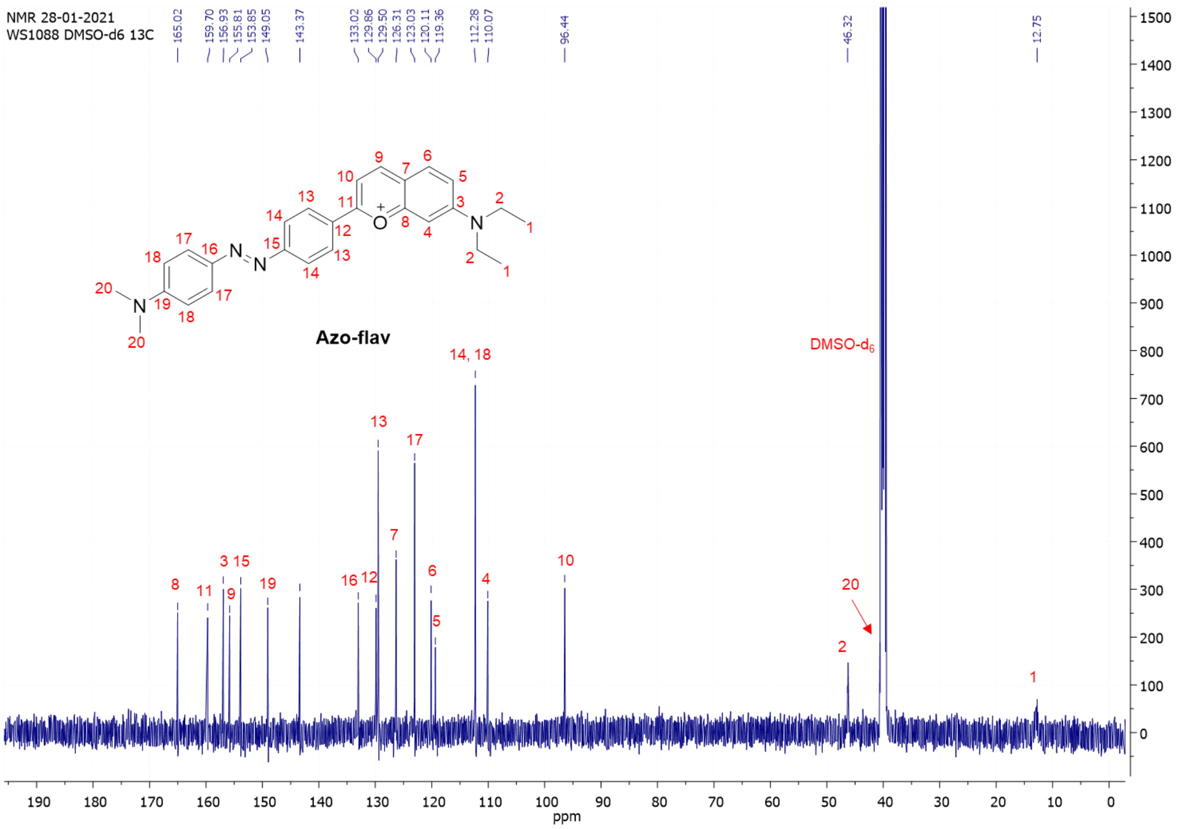

^{13}C NMR spectra of AZO-flav in DMSO-d$_6$

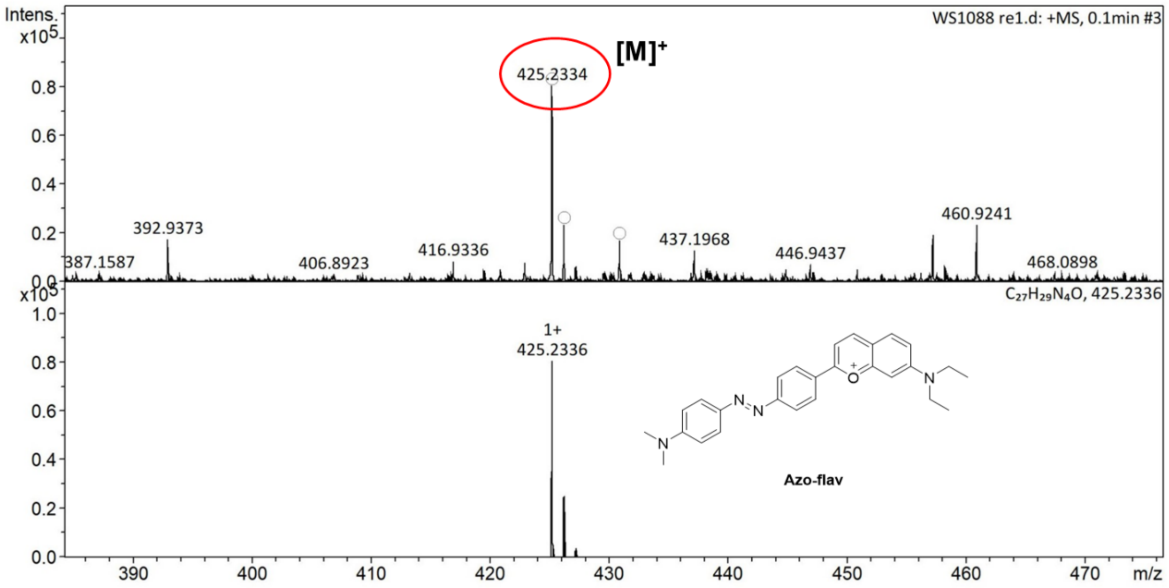

High resolution ESI^{+} spectra of AZO-flav

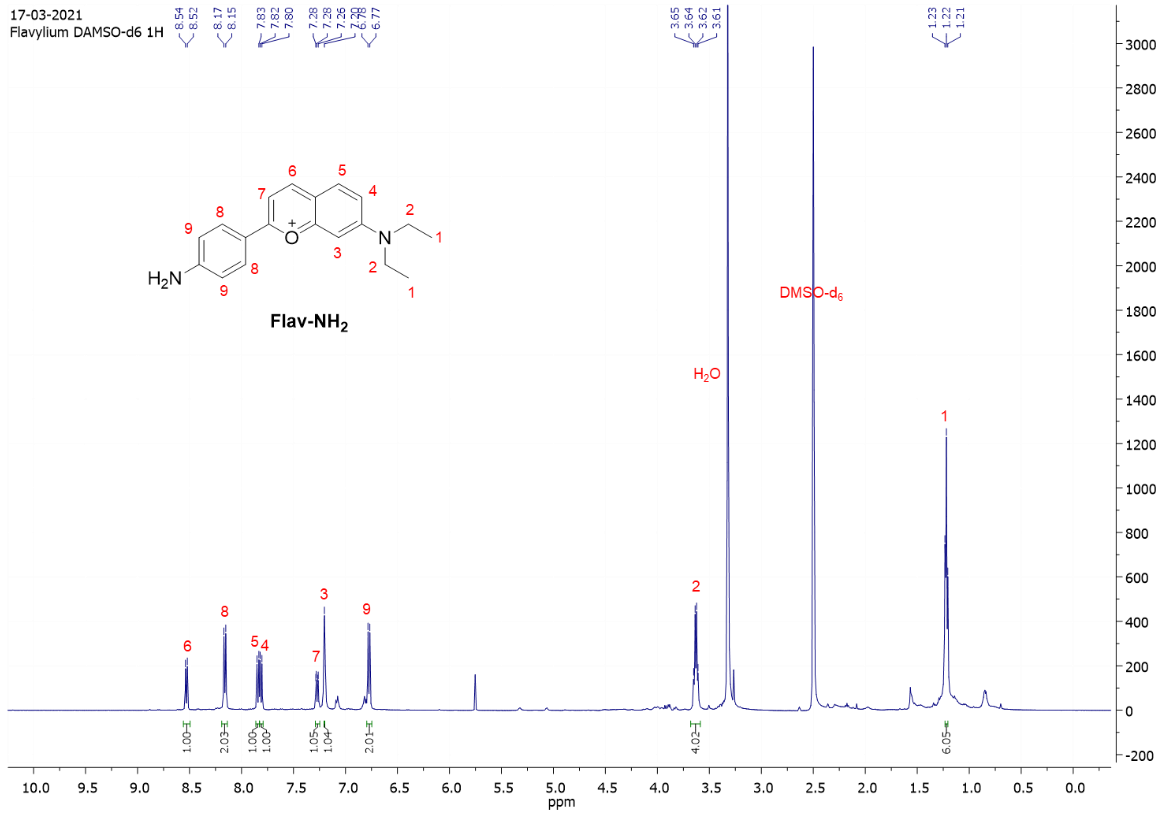

^{1}H NMR spectra of Flav-NH_2 in DMSO-d_6

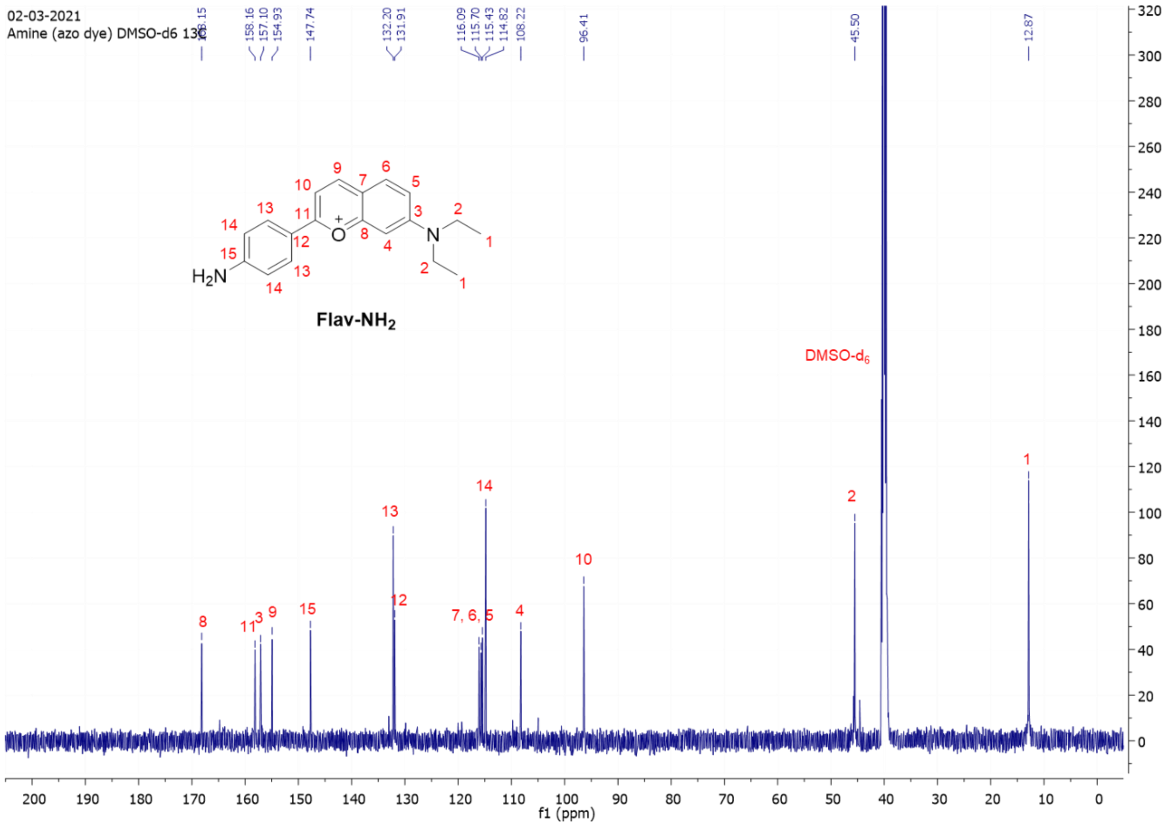

^{13}C NMR spectra of Flav-NH_2 in DMSO-d_6

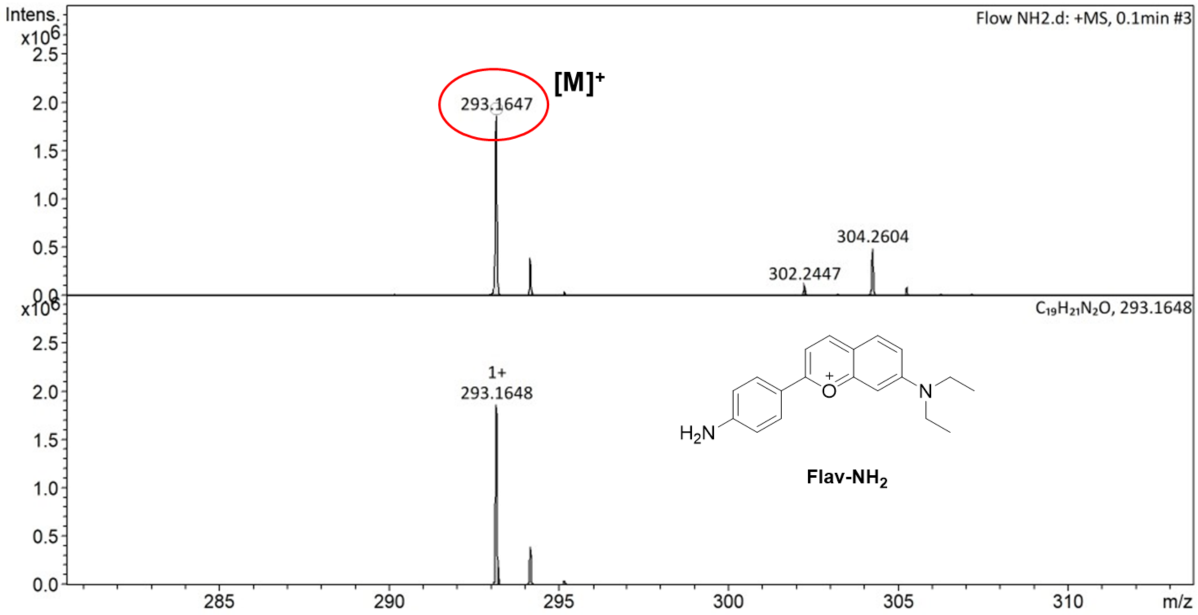

High resolution ESI$^+$ spectra of Flav-NH_2

Table S1. Comparison of AZO-Flav with commercially available hypoxia detection probes.

	**EF5 Alexa Fluor® 488**[1]	**BioTracker 520 Green Hypoxia Dye** [2]	**AZO-Flav**
**Structure**	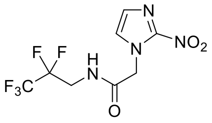	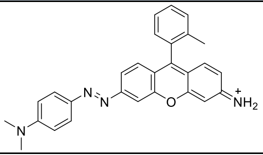	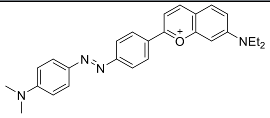
**Synthesis**	Substrates are noncommercial	5 Steps synthesis (see Scheme S1)	2 Steps synthesis with metal-free catalyzed reaction
**(λ_{ex}/ λ_{em})**	Depend on antibody	498/520nm	540/607nm
**Key applications**	Immunohistochemistry and Immunofluorescence	Cell based assay	Cell based assay
**Live cell fluorescent imaging**	Requirement of cell fixation followed by immunostaining	✓	✓
**Storage**	at -20°C	at -20°C	at 4-25°C
**How to detect?**	Binding to hypoxic cell and form adduct	Direct detection of O_2 level in living cell through enzymatic reduction	Direct detection of O_2 level in living cell through enzymatic reduction
**Electrical properties**	Uncharged, favor lipophilic	Charged	Charged, favor lipophilic (*N,N*-dimethylamino group)
**Photo stability**	Stable	Light sensitive (from SDS) but no report in the paper	Stable
**pH stability**	No data	Stable	Stable
**Limit of detection**	No data	No data	0.4 µM
**Minimum dose cellular uptake (cell line)**	No data	0.5 µM	5.0 µM
**O_2 concentration responded**	1%	5%	5%

Table 2. Comparison of AZO-Flav with a reported protein-based sensor.

	**Iglesias, P. et al.[3]**	**This work**
**Types of sensors**	Protein-based fluorescent biosensor	Activity-based fluorescent sensor
**Technique for synthesis**	Site-specific chemical modification of protein framework with synthetic fluorophore	Fully chemical synthesis (2 steps)
**Pathway**	Oxygen-dependent degradation of HIF-1α	Oxygen-dependent reduction of AZO-Flav
**Advantages**	High stability, low immunogenicity, good bio-distribution	High stability, selectivity, sensitivity and suitable for tissues and animal applications
**Limitations**	Applying to tissues and animals is difficult	Interference effect from local environment

Table S3. The structures and O_2 responses of azo-based probes

**Probes**	**O_2-concentration responded**	**Rate**	**Conditions**	**Ref.**
Cy-Azo	1% in HepG2 and HCT116 cells	reach the plateau at about 20 min	Azo reductase = 0.4 μg/mL; [NADPH]= 100 μM	[4]
NR-Azo	No report	reach the plateau at about 22 min	Mouse liver microsomes = 50 μg/mL; [NADPH]= 100 μM	[5]
Azo-Cy	5% in A549 cells	reach the plateau at about 4 min	Cytochrome P450 reductase = 1 U/mL; [NADPH]= 0.1 mM	[6]
Azo-DCM	10% in A549 cells	reach the plateau after mixed 7.5 min	Cytochrome P450 reductase = 1 U/mL; [NADPH]= 1 mM;	[7]
AzP1	3% in HeLa cells	reach the plateau after mixed 80 min	Rat liver microsomes = 200 μg/ml; [NADPH]= 0.1 mM	[8]
QCy5	1% in MCF-7 cells	reach the plateau at about 10 min	Rat liver microsomes = 50 μg/3ml; [NADPH]= 50 μM	[9]
HYPOX-3	1% in R28 cells	-	-	[10]
SR101-NaphtNH$_2$-Hyp-sulfobetaine	1% in A549 cells	Over 6 min	rat liver microsomes = 226 mg/3 mL; [NADPH]= 50 μM	[11]
MAR	5% in A549 cells	About 11 min	rat liver microsomes = 226 mg/3 mL; [NADPH]= 50 μM	[2]
HP	10% in HeLa cells	reach the plateau at about 7.5 min	Cytochrome P450 reductase = 1 U/mL; [NADPH]= 100 μM	[12]
Hyp-Ly	1% in HepG2 cells	reach the plateau at about 90 min	rat liver microsomes = 100 μg/mL; [CTAB]= 1mM	[13]
AZO-Flav	5% in HepG2 cells	reach the plateau at 2 min	*Ec*FldR reductase = 2 μM; [NADPH]= 50 μM	**This work**

3. Spectroscopic and methods

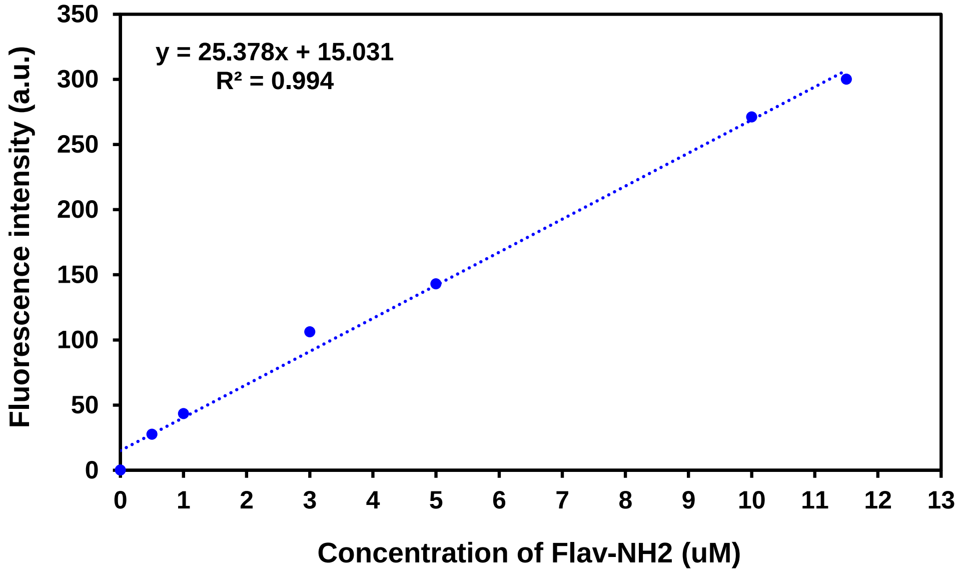

Figure S1. Calibration curve of Flav-NH_2 (λ_{ex} = 540 nm and λ_{em} = 607 nm).

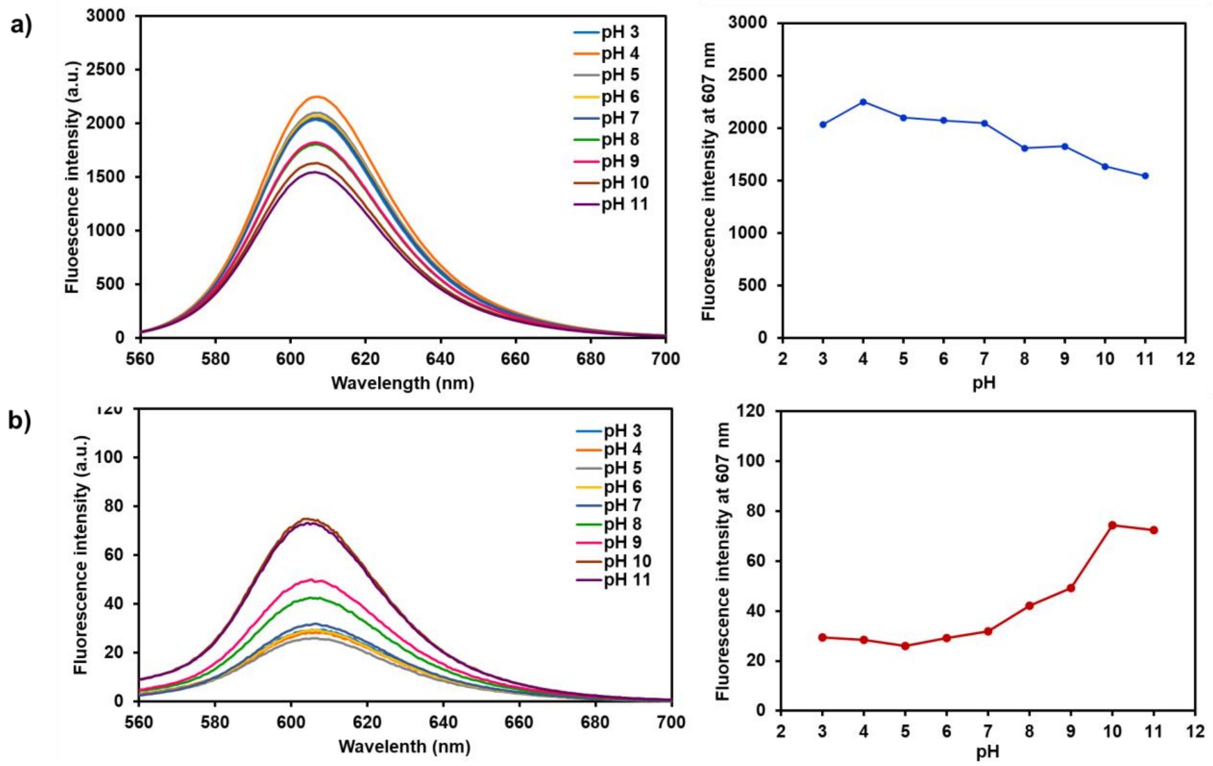

Figure S2. pH effect in 100 mM phosphate buffer at pH = 3, 4, 5, 6, 7, 8, 9, 10, and 11 with λ_{ex} = 540 nm and λ_{em} = 607 nm. a) 10 μM of Flav-NH_2 and b) 10 μM of AZO-flav.

4. Enzymatic Assays

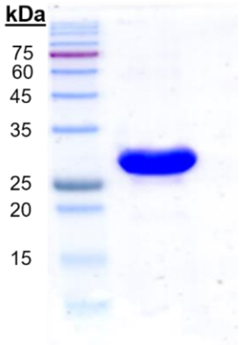

Figure S3. SDS-PAGE analysis of the purified *Ec*FldR.

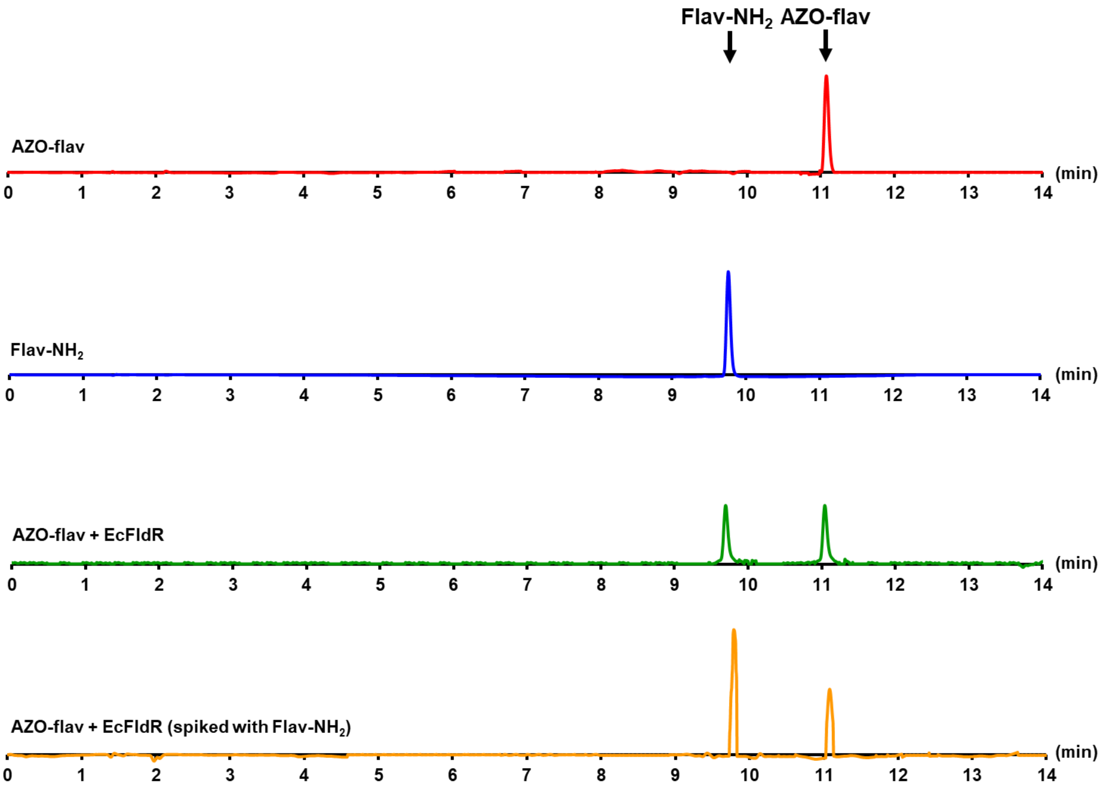

Figure S4. HPLC analysis of metabolism of AZO-Flav reacted with *Ec*FldR reductase. AZO-Flav (10 μM) and NADPH (50 μM) was treated with *Ec*FldR reductase (2 μM) for 5 min. HPLC profiles were detected by UV/Vis at 560 nm.

5. Biological studies

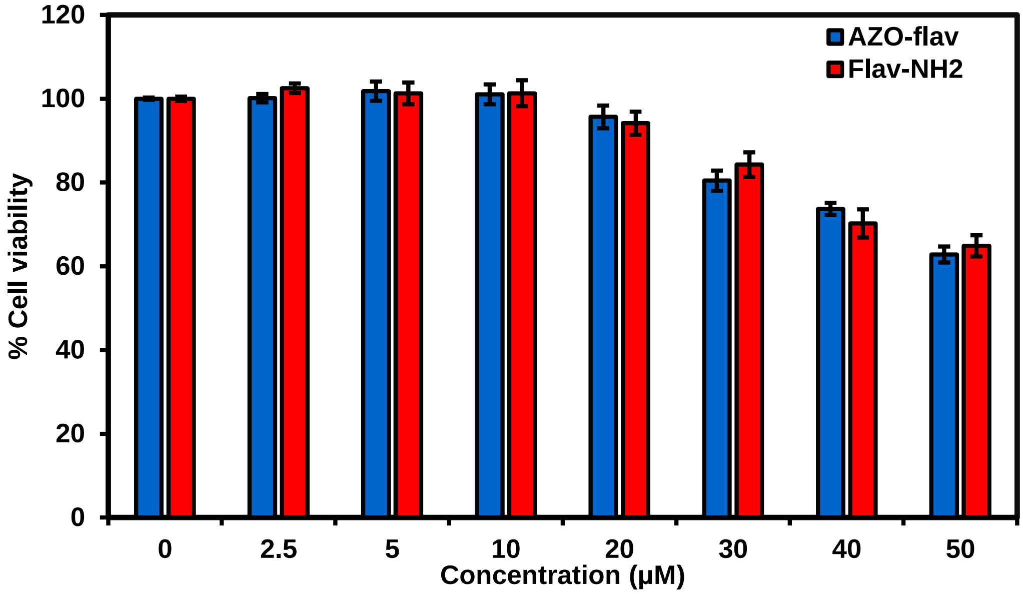

Figure S5. MTT assay of AZO-Flav and Flav-NH_2 in HepG2 at different concentration incubated for 24h.

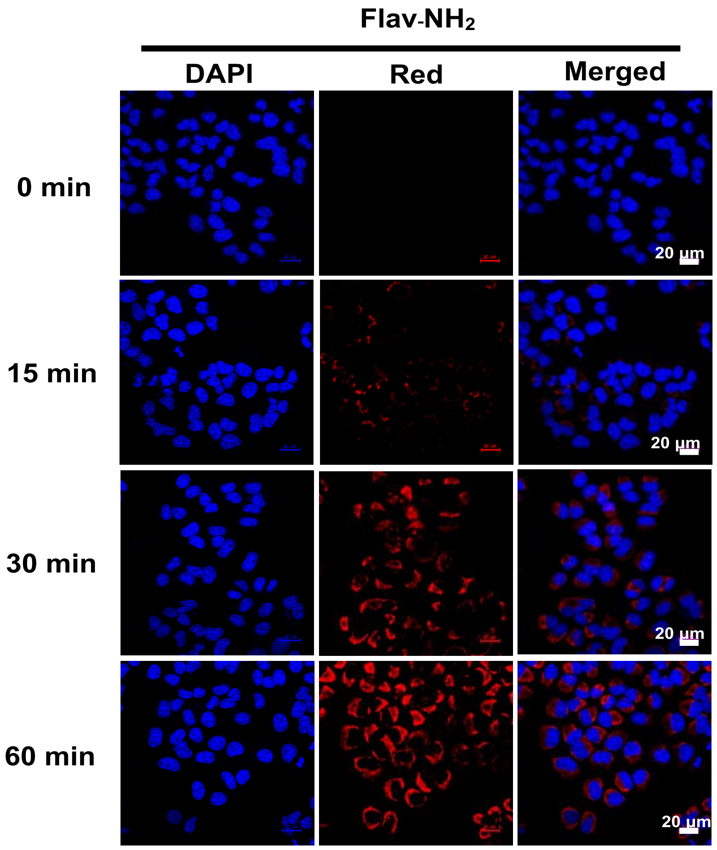

Figure S6. Time dependent cellular uptake of Flav-NH_2 incubated for 0, 15, 30, and 60 min.

Colocalization Study

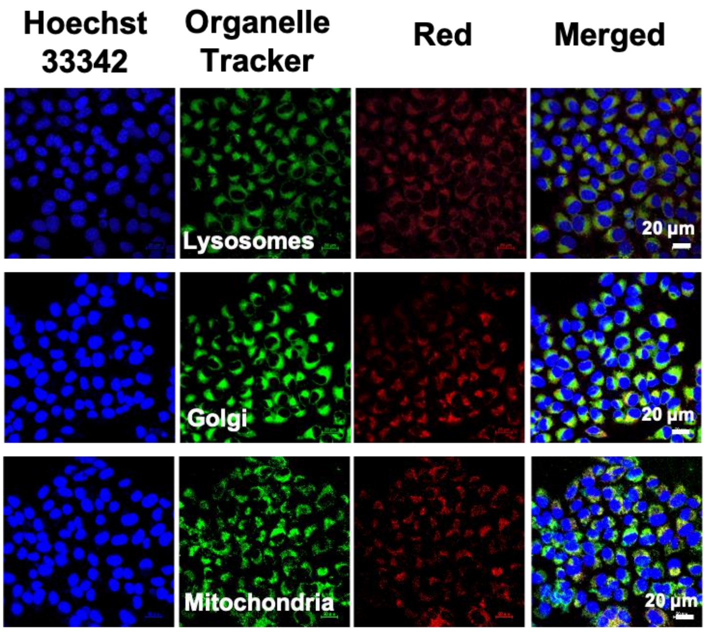

Figure S7. Confocal images of **AZO-Flav** incubated with hypoxic HepG2 cells and colocalized with sub-organelle trackers.

References

[1] C. J. Koch, *Methods Enzymol.* **2002**, *352*, 3-31.

[2] W. Piao, S. Tsuda, Y. Tanaka, S. Maeda, F. Liu, S. Takahashi, Y. Kushida, T. Komatsu, T. Ueno, T. Terai, T. Nakazawa, M. Uchiyama, K. Morokuma, T. Nagano, K. Hanaoka, *Angew. Chem. Int. Ed.* **2013**, *52*, 13028-13032.

[3] P. Iglesias, C. Penas, L. Barral-Cagiao, E. Pazos, J. A. Costoya, *Sci. Rep.* **2019**, *9*, 7117.

[4] Y. Tian, Y. Li, W.-L. Jiang, D.-Y. Zhou, J. Fei, C.-Y. Li, *Anal. Chem.* **2019**, *91*, 10901-10907.

[5] J. Huang, Y. Wu, F. Zeng, S. Wu, *Theranostics* **2019**, *9*, 7313-7324.

[6] C. Wang, S. Zhang, J. Huang, L. Cui, J. Hu, S. Tan, *RSC Adv.* **2019**, *9*, 21572-21577.

[7] L. Cui, Y. Shi, S. Zhang, L. Yan, H. Zhang, Z. Tian, Y. Gu, T. Guo, J. Huang, *Dyes and Pigments* **2017**, *139*, 587-592.

[8] Y. Zhou, M. Maiti, A. Sharma, M. Won, L. Yu, L. X. Miao, J. Shin, A. Podder, K. N. Bobba, J. Han, S. Bhuniya, J. S. Kim, *J. Control Release* **2018**, *288*, 14-22.

[9] K. Kiyose, K. Hanaoka, D. Oushiki, T. Nakamura, M. Kajimura, M. Suematsu, H. Nishimatsu, T. Yamane, T. Terai, Y. Hirata, T. Nagano, *J. Am. Chem. Soc.* **2010**, *132*, 15846-15848.

[10] M. I. Uddin, S. M. Evans, J. R. Craft, L. J. Marnett, M. J. Uddin, A. Jayagopal, *ACS Med. Chem. Lett.* **2015**, *6*, 445-449.

[11] A. Chevalier, W. Piao, K. Hanaoka, T. Nagano, P.-Y. Renard, A. Romieu, *Methods Appl. Fluoresc.* **2015**, *3*, 044004.

[12] Q. Cai, T. Yu, W. Zhu, Y. Xu, X. Qian, *Chem. Commun.* **2015**, *51*, 14739-14741.
[13] S. Luo, Y. Liu, F. Wang, Q. Fei, B. Shi, J. An, C. Zhao, C.-H. Tung, *Analyst* **2016**, *141*, 2879-2882.
